# Does the direct effect of friction increase continuously with absolute temperature?

**DOI:** 10.1073/pnas.2405111121

**Published:** 2024-10-10

**Authors:** Sylvain Barbot

**Affiliations:** ^a^Department of Earth Sciences, University of Southern California, Los Angeles, CA 90089-0740

**Keywords:** friction, fault mechanics

## Abstract

Constitutive models of fault friction form the basis of physics-based simulations of seismic activity. A generally accepted framework for the slip-rate and state dependence of friction involves a thermally activated process, whereby the probability of slip along microasperities adheres to an Arrhenius law. This model, which has become widely adopted among experimentalists and theoreticians, predicts a continuous increase of the direct effect with absolute temperature, but is it observed experimentally? Leveraging comprehensive laboratory data across diverse hydrothermal, barometric, and lithological conditions, we demonstrate that, contrary to the classical view, the direct effect for a given deformation mechanism remains largely temperature-independent. Instead, the incremental shifts in the direct effect often coincide with the brittle to semi-brittle transition, across which distinct deformation mechanisms operate. These considerations challenge the validity of the classical model. Realistic constitutive laws for rock failure within the lithosphere must incorporate the contributions of multiple deformation mechanisms within active fault zones.

## Significance

Establishing a constitutive law governing fault friction is a stepping stone for advancing physics-based predictions of the seismic cycle and associated hazards. A commonly accepted explanation for the slip-rate and state dependency of friction is rooted in the thermal activation of slip at microasperities that form the actual area of contact. The model predicts a continuous increase of the parameter regulating the direct influence of velocity on the frictional resistance. However, this perspective conflicts with numerous laboratory observations portraying a different behavior. Specifically, the direct effect parameter is relatively unaffected by temperature up to the brittle to semi-brittle transition. Addressing these disparities and capturing realistic rock behavior within constitutive laws requires considering the contribution of multiple deformation mechanisms.

The mechanics of earthquakes and faulting within the lithosphere are controlled by the constitutive behavior of rocks (1–4). Early experimental and theoretical studies (5–8) have motivated the formulation of the slip-rate and state-dependent friction law

[1] $$\begin{array}{c} \mu =\mu_{0}+a\ln\frac{V}{V_{0}}+b\ln\frac{\theta V_{0}}{L}\;, \end{array}$$

where V and $V_{0}$ are the slip-rate and a reference value, respectively, θ is the state variable representing the age of contact, which is subject to an evolution law, with the characteristic age $L/V_{0}$, and a and b control the slip-rate and state dependence, respectively. The empirical formulation explains many interconnected aspects of fault dynamics during the seismic cycle, including the propagation of earthquakes (9–14), the emergence of slow-slip events and low-frequency earthquakes (15–21), and the correlative variability in rupture style and recurrence patterns (22–24).

The empirical formulation of Eq. **1** finds a physical basis in the thermal activation of slip at microasperities that form the real area of contact (25–39), predicting a continuous rise of the direct effect parameter with absolute temperature

[2] $$\begin{array}{c} a=\frac{\mathrm{RT}}{\Omega \chi_{n}}\;, \end{array}$$

where R is the universal gas constant, Ω is the molar activation volume, and $\chi_{n}$ is the indentation hardness. Recent efforts to incorporate the evolution of the constitutive parameters with surrounding physical conditions often base their formulation on the same starting assumptions with additional considerations (40–49). Despite its significant relevance for earthquake science, the predictive capabilities of (Eq. **2**) have not been thoroughly assessed, with great implications for the body of work built upon it.

In this study, we use laboratory measurements from velocity-step experiments to test the validity of (Eq. **2**) and its underlying assumptions. First, we describe in detail the theory behind (Eq. **1**). We then present a large number of laboratory data that document the temperature dependence of the direct effect parameter a from room temperature up to 600 ^°^C in experiments where a single deformation mechanism is in effect. In most cases, we can reject the hypothesis that a increases continuously with absolute temperature following (Eq. **2**), which calls for a thorough reassessment of common assumptions invoked in physical models of rock friction. We then describe how considering multiple deformation mechanisms operating at different slip-rate, pressure, and temperature regimes can explain the behavior of rocks in various laboratory settings.

## Reference Model

We describe the physical assumptions that form the basis of (Eq. **1**) following previous work (34, 38, 50), which we refer to as the reference model. Due to the roughness of natural surfaces, a frictional interface is supported by a lattice of microasperities (33, 51–53). The frictional resistance results from the strength of a microasperity and the contact density

[3] $$\begin{array}{c} \tau =\chi (V)A(\theta )\;, \end{array}$$

where the plowing hardness χ is a function of slip-rate and the real area of contact density A is a function of the age of contact. Based on laboratory observations and theory (31, 51, 52, 54), the real area of contact density ages as follows

[4] $$\begin{array}{c} A=\frac{\sigma }{\chi_{n}}(1+m\ln\frac{\theta V_{0}}{L})\;, \end{array}$$

where σ is the effective normal stress, $\chi_{n}$ is the indentation hardness, and m≪1 is a constitutive parameter. Microasperity creep is thermally activated (25, 26) with a potential energy barrier modified by the influence of shear stress and random thermal fluctuations, leading to (ref. 29)

[5] $$\begin{array}{c} V=V_{0}\exp[-\frac{E-\chi \Omega }{\mathrm{RT}}]\;, \end{array}$$

where E and Ω are the energy and volume of activation for a mole of constituent. Connecting Eqs. **3**–**5**, we get the slip-rate and state-dependent friction law

[6] $$\begin{array}{c} \mu =\frac{\tau }{\sigma }=\frac{1}{\chi_{n}}(\frac{E}{\Omega }+\frac{\mathrm{RT}}{\Omega }\ln\frac{V}{V_{0}})(1+m\ln\frac{\theta V_{0}}{L})\;. \end{array}$$

Neglecting the squares of logarithms that play a negligible role, we get Eqs. **1** and **2** with $\mu_{0}=E/\Omega \chi_{n}$ and $b=m\mu_{0}$. The evolution of the frictional resistance during seismic cycles is based on an evolution law for the age of contact (8), often given by the aging law in isobaric, isothermal conditions

[7] $$\begin{array}{c} \dot{\theta }=1-\frac{V\theta }{L}\;, \end{array}$$

or the slip law,

[8] $$\begin{array}{c} \dot{\theta }=-\frac{V\theta }{L}\ln\frac{V\theta }{L}\;, \end{array}$$

which, despite producing different evolutionary effects, converge to the same steady-state solution (55–59). With this formulation, the choice of the evolution law has no bearing on the direct effect, which occurs at constant state.

The use of logarithms in (Eq. **1**) produces an unbounded negative friction coefficient for sufficiently low velocities that forbids stationary contact. This problem is typically addressed by considering the exponentially decaying probability of backward motion at low shear stress and a state-dependent activation energy, leading to a modified friction law (29, 30) (*SIAppendix*, section 3)

[9] $$\begin{array}{c} \mu =a\;\sinh^{-1}[\frac{V}{2V_{0}}\exp(\frac{\mu_{0}+b\ln\frac{\theta V_{0}}{L}}{a})]\;. \end{array}$$

The above formulation is widely used in numerical simulations of the seismic cycle (10–12, 15, 50, 60–63). However, the validity of (Eq. **9**) hinges on the applicability of the physical assumptions described above. The reference model makes predictions that can be tested with laboratory observations, such as a monotonic increase of the direct effect parameter with absolute temperature. Is it confirmed experimentally?

## Verification

We inspect laboratory observations from velocity-step experiments encompassing triaxial, rotary shear, and direct shear apparatuses that place strong constraints on the constitutive behavior of rocks in various tectonic contexts. In many cases, laboratory constraints show some scatter, typically due to the dependence of the direct effect parameter on total strain, undocumented fluctuations in pore-fluid and confining pressure and temperature, and trade-offs with other variables during parameter estimation, among other factors. For each experiment, we test the null hypothesis that the direct effect increases monotonically with absolute temperature, as predicted by (Eq. **2**) in the reference model, against the alternative hypothesis where the direct effect parameter is a constant independent of temperature. We conduct an F-test that compares the residuals between the laboratory measurements and the prediction of (Eq. **2**) after best-fitting the product $\Omega \chi_{n}$ using least-squares associated with the null hypothesis and the residuals after removing the mean value corresponding to the alternative hypothesis. In all cases, we check that the residuals between laboratory observations and model predictions can be well represented by a normal distribution. Using an Anderson-Darling goodness-of-fit test, this hypothesis cannot be rejected at the 5% significance level.

**Fig. 1.**
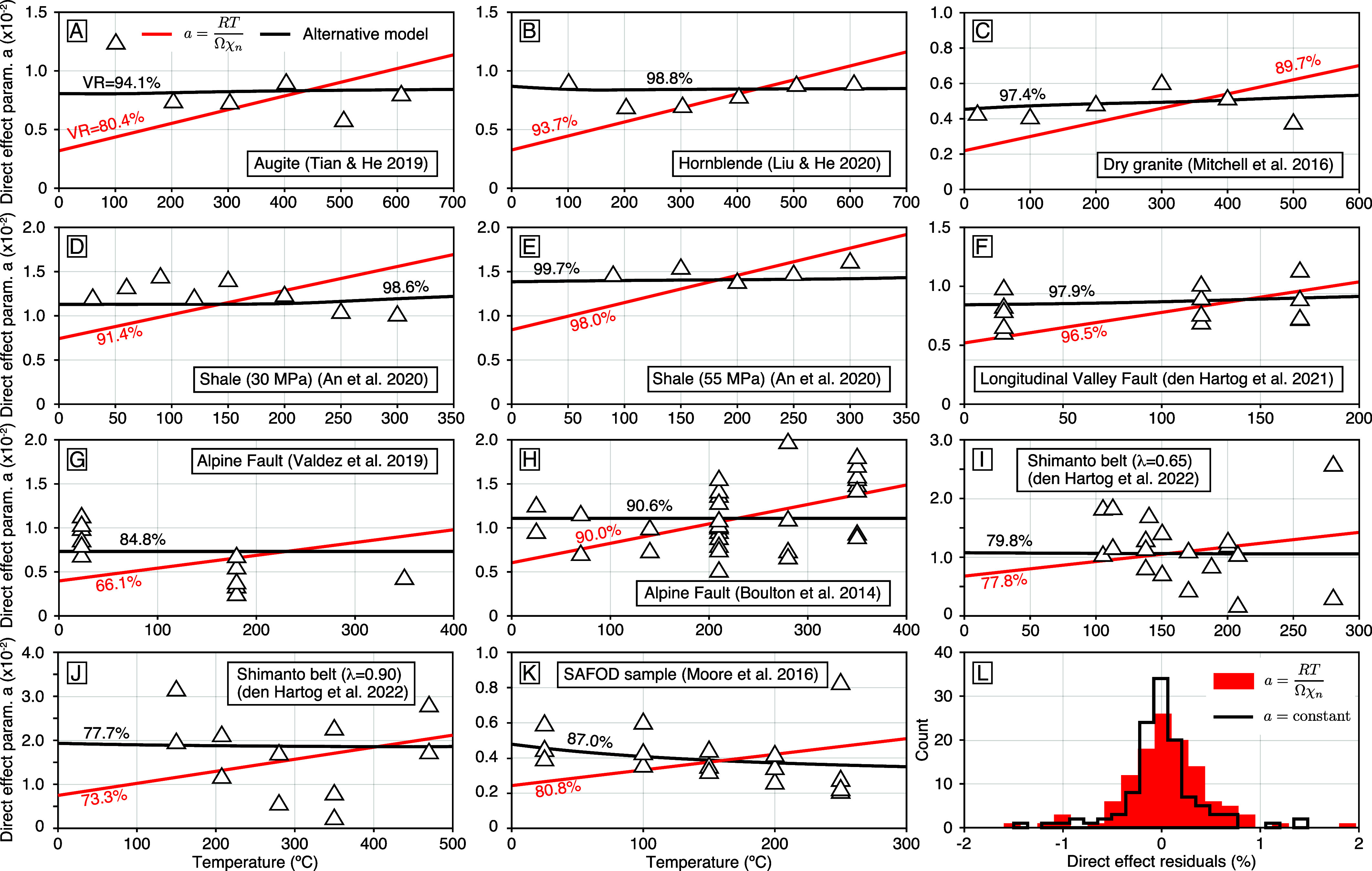
Evolution of the direct effect parameter as a function of temperature for predominantly a single deformation mechanism for (*A*) augite (64) and (*B*) hornblende (65) in triaxial shear at ∼100 MPa normal stress, (*C*) dry Westerly granite in single direct shear at 5 MPa (66), (*D* and *E*) Sichuan Basin shale at 30 MPa and 55 MPa, respectively, in a triaxial setting (67); followed by natural gouge from (*F*) the Longitudinal Valley Fault in Taiwan (sample LVF34) (68), the Alpine Fault in New Zealand at (*G*) a constant effective normal stress of 80 MPa (69) and (*H*) varying normal stress (70); from the Shimanto belt in Japan at (*I*) low and (*J*) high pore-fluid pressure; followed by (*K*) natural samples from the San Andreas Fault Observatory at Depth (71). The variance reduction (VR =$1-r_{k}r_{k}/d_{k}d_{k}$, where $r_{k}r_{k}$ and $d_{k}d_{k}$ indicate the sum of the squares of the residuals and laboratory measurements, respectively) of the reference and alternative models with a constant direct effect parameter is written in black and red text, respectively. The model predictions for the reference model and of the alternative model with a single deformation mechanism are shown with the red and black lines, respectively. (*L*) Histogram of residuals with the reference model (red) and histogram of residuals with the alternative model that assumes a constant value (solid black line).

In deformation experiments on augite gouge (Fig. 1*A*), the velocity steps conducted at an effective normal stress of 97MPa reveal a direct effect parameter between 6% and 12% with a weakly decreasing tendency from 100 ^°^C to 600 ^°^C (64). Assuming a monotonically increasing or constant value reduces the data by 80.4% and 94.1%, respectively, corresponding to reduced chi-squares of 4.2 and 1.3 when taking measurement uncertainties of 0.2%. By visual inspection, the reference model produces systematic residuals. Based on the F-test, we can reject the null hypothesis with 89% confidence. Experiments at similar barometric conditions on hornblende (Fig. 1*B*), show oscillations of the direct effect parameter between 7% and 9% from 100 ^°^C to 500 ^°^C (65). The reference model does not capture the overall trend of the observations. Based on the F-test, we can reject the null hypothesis at 95.3% confidence.

Experiments on dry Westerly granite gouge at 5 MPa normal stress in direct shear (66) show a fairly constant parameter for the direct effect of velocity between 1% and 9% from room temperature to 500 ^°^C (Fig. 1*C*). Similar measurements are obtained at 30 MPa normal stress (66). The reference and alternative models reduce the data by 89.7% and 97.4%, respectively, with reduced chi-squares of 1.2 and 0.2. We can reject the null hypothesis at 92.4% confidence. Experiments on Sichuan shale gouge conducted at effective confining pressure of 30 MPa and 55 MPa (67) also reveal fairly uniform values oscillating between 10% and 16% from room temperature to 300 ^°^C (Fig. 1 *D* and *E*). At each pressure, the variance reduction of the alternative model is superior. We can reject the null hypothesis at 98.6% and 95.9% confidence for the cases of 30 MPa and 55 MPa, respectively. Studies on natural gouge from a velocity-weakening segment of the Longitudinal Valley Fault in Taiwan (68) also reveal a fairly constant direct effect parameter from room temperature to 170 ^°^C (Fig. 1*F*). Based on these observations, we can reject the null hypothesis at the 81.6% confidence level. Measurements from other sections of the Longitudinal Valley Fault in similar physical conditions show comparable results (68).

Gouge extracted from the upper principal slip zone (PSZ-1) of the Alpine Fault Deep Fault Drilling Project (Fig. 1*G*) exhibits a direct effect parameter decaying from 11% to 4% from room temperature to 350 ^°^C at a constant effective normal stress of 80 MPa, including the effect of a 40 MPa pore-fluid pressure (69), firmly invalidating the reference model, which predicts an increase. Based on the F-test, the null hypothesis can be rejected with 90.0% confidence. Measurements conducted at various temperatures and confining pressures on Alpine Fault samples to mimic the effect of a geothermal gradient (70) show more scatter (Fig. 1*H*). In this case, the null hypothesis can only be rejected with only 54.8% confidence.

Gouge formed from natural samples of the Shimanto belt at low and high pore-fluid pressure (72) show much variability (Fig. 1 *I* and *J*). In both cases, however, the alternative model reduces the data more effectively. Based on the F-test, we can reject the null hypothesis with a 58.1% and 61.2% confidence level, respectively. Finally, we consider measurements from natural gouge extracted from the San Andreas Fault Observatory at Depth (71), which show a decreasing trend of the direct effect from room temperature to 250 ^°^C (Fig. 1*K*). We can reject the null hypothesis for these data with 77.3% confidence.

Overall, the data presented in Fig. 1 are better explained assuming a direct effect independent of temperature. Assuming a monotonically increasing direct effect produces more systematic residuals (Fig. 1*L*). Based on an F-test with the residuals of all samples, we can reject the null hypothesis with 93.3% confidence. These findings call for the abandonment of the reference model and prompt the reconsideration of its underlying physical assumptions.

## Alternative Model

**Fig. 2.**
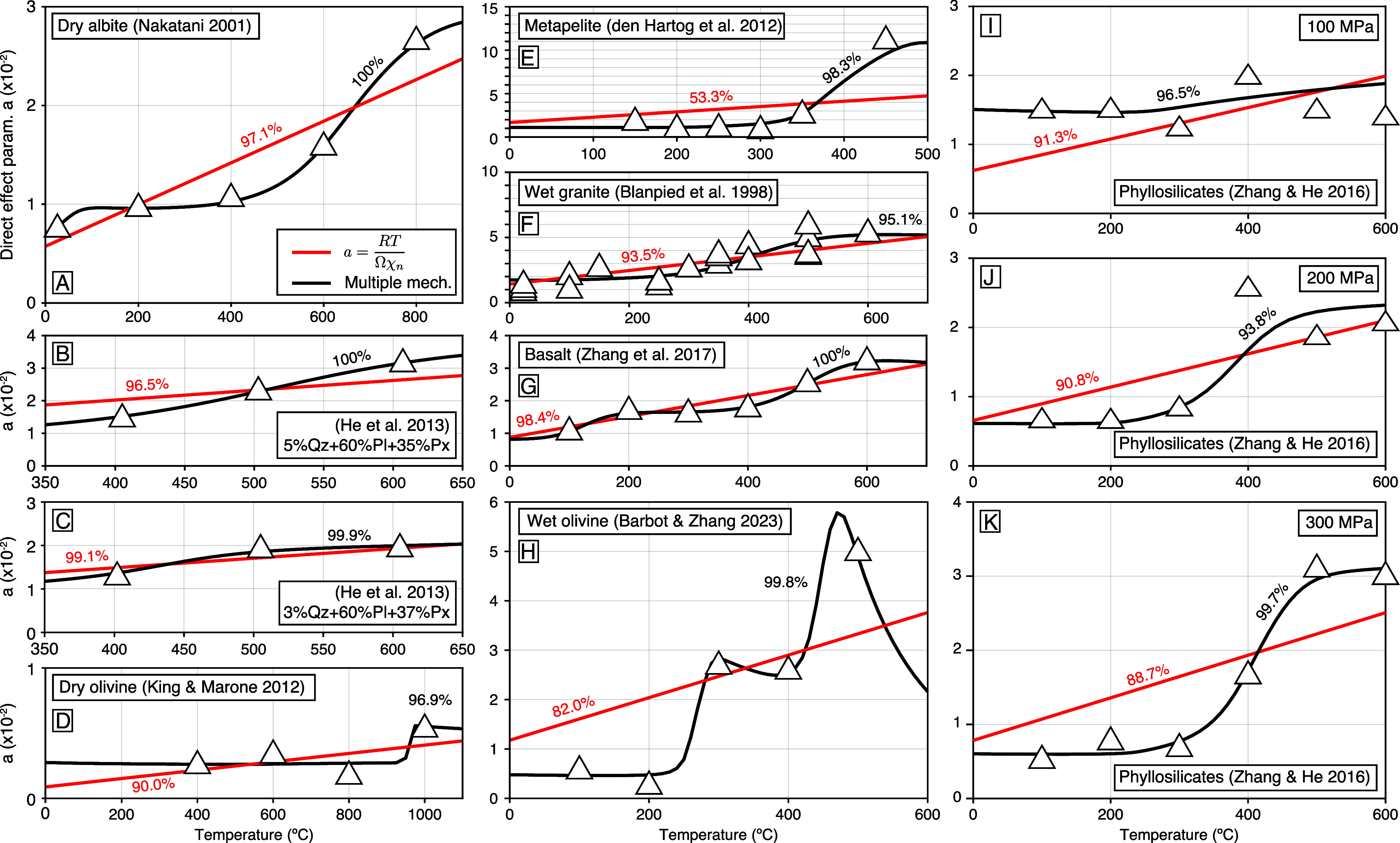
Evolution of the direct effect parameter in experimental conditions where multiple mechanisms operate at different temperatures. Cases of (*A*) dry albite gouge (35), (*B* and *C*) plagioclase and pyroxene gouge with various proportions of quartz (Qz) and pyroxene (Px) (77), (*D*) dry olivine gouge (78), (*E*) metapelite (79), (*F*) wet Westerly granite gouge under 400 MPa of effective normal stress (2, 80), (*G*) basalt gouge under 50 MPa effective normal stress (81), and (*H*) wet olivine gouge with an effective normal stress of 150 MPa (82). (*I*, *J*, and *K*) Evolution of the direct effect parameter on phyllosilicates with varying temperature and pressure for an effective normal stress of 100 MPa, 200 MPa, and 300 MPa, respectively, involving fluids pressurized at 30 MPa (83). The predictions of the reference model (red) and the alternative model with multiple deformation mechanisms (black) are shown in each case with the corresponding variance reduction.

While numerous laboratory observations suggest a limited temperature dependence of the direct effect, as depicted in Fig. 1, it is acknowledged that several other experiments demonstrate a significant increase in the direct effect with absolute temperature, as illustrated in Fig. 2. However, the increase is typically step-wise, suggesting the activation of distinct deformation mechanisms in specific temperature ranges. Recognizing this range of possible behaviors, we require an alternative model that affords different outcomes upon various parametric configurations.

We describe a physical framework for rock friction based on the contribution of multiple deformation mechanisms (54, 73–75) presented as a possible alternative to the reference model. The frictional resistance of natural surfaces is controlled by the real area of contact $A_{r}$, which is significantly smaller than the nominal area of contact $A_{0}$. The real area of contact density $A=A_{r}/A_{0}$ can be written (54, 73–75)

[10] $$\begin{array}{c} A=\frac{\sigma }{\chi_{n}}{\left(\frac{d}{d_{0}}\right)}^{\alpha }{\left(\frac{\sigma }{\sigma_{0}}\right)}^{-\beta }\;, \end{array}$$

where the size of contact d is a state variable subject to an evolution law and $d_{0}=1\;\mu$m is a fixed reference size, σ and $\sigma_{0}=10\;$MPa are the effective normal stress and a fixed reference value, respectively, and α≪1 and β≪1 are power exponents. At the macroscopic level, the yield strength emerges from the strength of microasperities and the real area of contact density,

[11] $$\begin{array}{c} \tau_{y}=A\chi \;, \end{array}$$

where, in contrast to the reference model, χ is assumed independent of velocity. Combining Eqs. **10** and **11**, only the ratio $\chi /\chi_{n}$ intervenes, which cancels any common dependence of temperature and velocity of material hardness. A slip-rate-, state-, normal-stress-, and temperature-dependent friction law can be obtained by assuming a thermally activated constitutive law for frictional sliding,

[12] $$\begin{array}{c} \frac{V}{V_{0}}=(\frac{\tau }{\tau_{y}}{)}^{n_{1}}\exp[-\frac{Q_{1}}{R}(\frac{1}{T}-\frac{1}{{\overset{¯}{T}}_{1}})]\;, \end{array}$$

where V is the amplitude of the slip-rate vector and τ is the amplitude of the shear traction vector, both nonnegative quantities. The direction of slip is coeval with the direction of the traction vector and the absence of traction implies stationary contact without further regularization. The thermodynamic parameters $Q_{1}$ and ${\overset{¯}{T}}_{1}$ are the energy and temperature of activation, respectively. The power-law exponent $n_{1}\gg 1$ depends on the rock type and texture at the interface. However, deformation may be accommodated by various mechanisms of deformation, such as granular flow, cataclasis, Riedel fractures, and localized plasticity within the active shear zone. To capture the evolution of the direct effect parameter associated with different creep mechanisms, we may consider the sum of the individual strain-rates,

[13] $$\begin{array}{c} \begin{array}{cc} \frac{V}{V_{0}} & =\sum_{k=1}^{M}(\frac{\tau }{\tau_{y}}{)}^{n_{k}}\exp[-\frac{Q_{k}}{R}(\frac{1}{T}-\frac{1}{{\overset{¯}{T}}_{k}})]\\ & \;+\sum_{k=M+1}^{M+P}(\frac{\tau }{\tau_{0}}{)}^{n_{k}}\exp[-\frac{Q_{k}}{R}(\frac{1}{T}-\frac{1}{{\overset{¯}{T}}_{k}})]\;, \end{array} \end{array}$$

combining M brittle and P semi-brittle or ductile mechanisms of deformation, where $\tau_{0}$ is a reference stress for plastic deformation. The semi-brittle and ductile mechanisms are not associated with a state variable and are characterized by a rate-dependent strength.

The constitutive behavior is completed by an evolution law that captures the flattening of contact junctions and the compaction of the gouge layer possibly by pressure-solution creep, viscoelastic collapse, and subcritical crack growth (73). The evolution law for the size of contact can be given by a formulation compatible with the aging law but augmented to incorporate the effects of temperature and normal stress (33, 40, 41) on N healing mechanisms (73–76)

[14] $$\begin{array}{c} \frac{\dot{d}}{d}=\sum_{k=1}^{N}\frac{G_{k}}{p_{k}d^{p_{k}}}(\frac{\sigma }{\sigma_{0}}{)}^{q_{k}}\exp[-\frac{H_{k}}{R}(\frac{1}{T}-\frac{1}{T_{k}})]-\frac{\lambda V}{2h} \end{array}$$

or another formulation compatible with the slip law

[15] $$\begin{array}{c} \frac{\dot{d}}{d}=\frac{\lambda V}{2h}\ln\{\frac{2h}{\lambda V}\sum_{k=1}^{N}\frac{G_{k}}{p_{k}d^{p_{k}}}(\frac{\sigma }{\sigma_{0}}{)}^{q_{k}}\exp[-\frac{H_{k}}{R}(\frac{1}{T}-\frac{1}{T_{k}})]\}, \end{array}$$

where $G_{k}=(1$µm${)}^{p_{k}}$/s is a reference rate of growth with the asperity-size and normal-stress power exponents $p_{k}$ and $q_{k}$, $H_{k}$ is the activation energy for healing, 1/λ is a characteristic strain controlling the weakening distance, and V/2h is the average strain-rate within the shear zone. Using Eqs. **14** or **15** does not impact the direct effect of velocity, which occurs at constant state, pressure, and temperature.

As all deformation mechanisms are thermally activated, one is likely to predominate in a specific temperature, normal stress, and slip-rate regime. In any condition, however, an effective power-law exponent can be defined as

[16] $$\begin{array}{c} n=\frac{\partial \ln V}{\partial \ln\tau }\;. \end{array}$$

Even though the direct effect parameter is not explicitly included in (Eq. **13**), it can readily be obtained through

[17] $$\begin{array}{c} a=\frac{\partial \mu }{\partial \ln V}=\frac{\mu }{n}\;, \end{array}$$

where the friction coefficient is defined as μ=∂τ/∂σ, potentially a function of normal stress (75). A single deformation mechanism (e.g., using M=1 and P=0 in (Eq. **13**)) accounts for all the experimental data shown in Fig. 1, as shown in prior work (54, 73). The thermodynamic parameters encompass stress power exponents $n_{1}=35\;\;\text{to}\;150$ and activation energies $Q_{1}=6\;\;\text{to}\;60$ kJ/mol (*SI Appendix*, Table S1), predicting a nearly uniform direct effect across the range of experimental conditions for the majority of samples. Consequently, the alternative model outperforms the reference model in all cases.

We now consider laboratory data that exhibit significant variations in the direct effect parameter (Fig. 2), requiring 2 or 3 deformation mechanisms with power-law exponents in the range n=7to110 and activation energies for the direct effect of temperature between 30 kJ/mol and 450 kJ/mol (*SI Appendix*, Table S2). All the constitutive parameters can be inferred within uncertainties and possible tradeoffs from the evolution of frictional strength during velocity-steps conducted at various temperatures, except for the width of the shear zone h, which results from the experimental design. The mechanical data for albite from a double-direct shear apparatus under a constant normal stress of 20 MPa in dry conditions from room temperature to 800 ^°^C can be explained using three deformation mechanisms, perfectly capturing the step-wise transition from a = 1% between 100 ^°^C and 500 ^°^C to a = 2.6% at 800 ^°^C. Similarly, three deformation mechanisms capture the evolution of the direct effect parameter for basalt under effective normal stress of 50 MPa with 100 MPa pore-fluid pressure with temperature from 100 ^°^C to 600 ^°^C (81), and for olivine under effective normal stress of 250MPa with a pore-fluid pressure of 100 MPa (82).

In contrast, using two deformation mechanisms (e.g., using M=1 and P=1 in (Eq. **13**)) is sufficient to explain the direct effect parameter for mixtures of plagioclase and pyroxene with 3% and 5% quartz under an effective normal stress of 200 MPa from 100 ^°^C to 600 ^°^C (77), for dry olivine in a triaxial setting with a confining pressure of 100 MPa from 400 ^°^C to 1,000 ^°^C (78), for metapelite in rotary shear experiments under 170 MPa confining pressure and 100 MPa pore-fluid pressure and temperatures of 150 ^°^C to 500 ^°^C (79), for Westerly granite under 400 MPa of effective normal stress accounting for 100 MPa of pore-fluid pressure from room temperature to 600 ^°^C (2, 80), and for phyllosilicates under 100 MPa, 200 MPa, and 300 MPa confining pressure with 30 MPa pore-fluid pressure up to 600 ^°^C (83). The alternative model systematically outperforms the reference model, often offering perfect fit to the data.

## Discussion

**Fig. 3.**
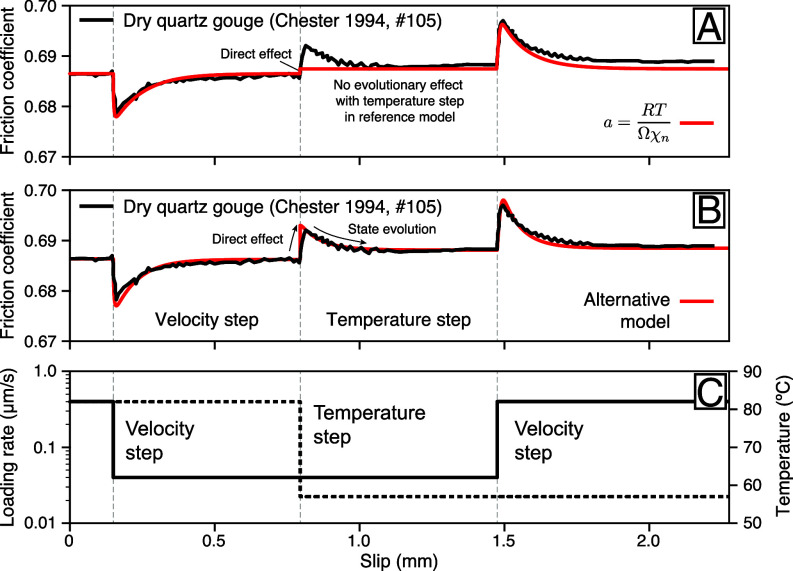
Constraints on velocity and temperature steps on the thermodynamic properties of gouge friction. (*A*) Laboratory observations for dry quartz gouge (41) (black line) and best-fitting predictions based on the reference model (red line). The reference model only produces a direct effect upon temperature change, but no transient evolution. (*B*) Laboratory observations (black line) and predictions of the alternative model with (Eq. **15**) using a single deformation mechanism (red line), capturing the direct and transient effects upon velocity and temperature steps. (*C*) Imposed velocity and temperature.

Constitutive friction laws serve as the foundational framework for physics-based seismic cycle simulations and seismic hazard analysis based on stress interactions. Given their extensive applications in earthquake science, there is a critical need for a quantitative understanding of rock failure with predictive power. Our analysis demonstrates the inadequacy of the reference model in elucidating many experimental findings across a spectrum of lithology representative of various tectonic and physiographic contexts of interest for induced and natural seismicity. A refined reference model with evolving activation volume and/or indentation hardness as a function of temperature and velocity in (Eq. **2**) may satisfy the constraints shown in Figs. 1 and 2. However, the functional form of the reference model is inadequate to explain the mechanical response to temperature perturbations (Fig. 3). Temperature-step experiments on dry quartz gouge under 20 MPa confining pressure at low slip-rate showcase a direct effect followed by a transient relaxation (41). The reference model predicts only a direct effect through (Eq. **2**) and it is generally not possible to explain velocity steps and temperature steps with a single set of constitutive parameters. The velocity step on dry quartz gouge at 82 ^°^C constrains the direct effect parameter a=0.0042, immediately providing $\Omega \chi_{n}=700\;$kJ/mol, an unlikely large activation energy. The formulation of the reference model is generally ill-posed because arbitrarily large changes of temperature inflict no variation of friction when $V=V_{0}$. In contrast, the alternative model captures the mechanical response upon the velocity and temperature steps consistently assuming a single deformation mechanism with $n_{1}=144$ and $Q_{1}=55\;$kJ/mol (*SI Appendix*, Table S3).

Given the weight of evidence, we can discard the specific physical assumptions that underlie the reference model as well as the friction laws of Eqs. **1** and **9**. In general, the empirical law of (Eq. **1**) derives from the Taylor series expansion of a currently unknown function in terms of the logarithm of the dynamic variables, which may encompass slip-rate, state, normal stress, and other variables (*SI Appendix*, section 4). As a result, (Eq. **1**) is only valid at constant coefficients for restricted barometric and hydrothermal conditions and for a limited range of slip-rates. A candidate function that explains more, but not all, conditions is given by (Eq. **13**).

**Fig. 4.**
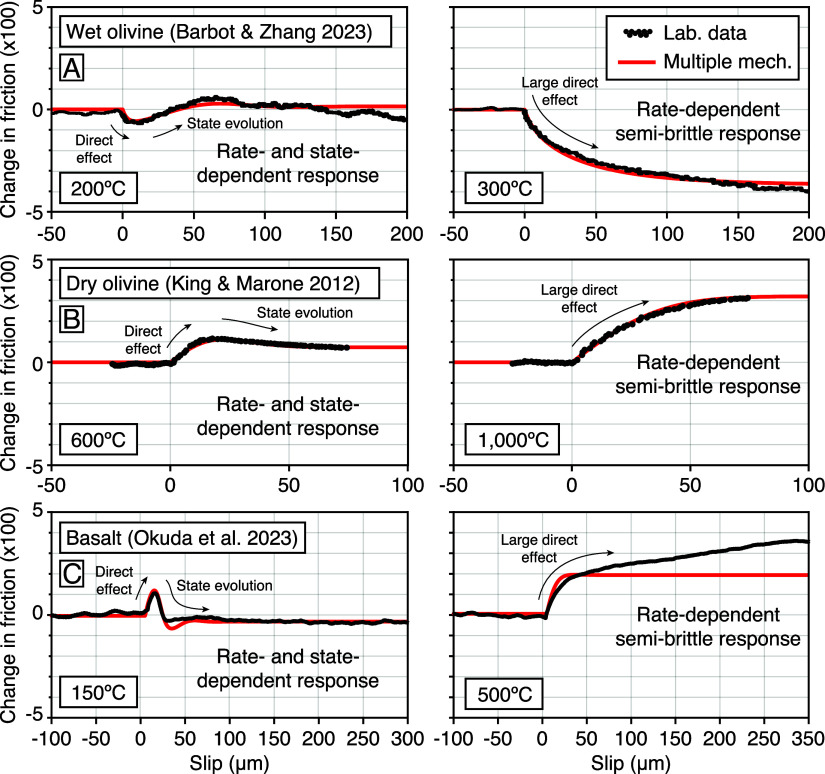
Mechanical response to velocity steps involving different deformation mechanisms. (*A*) Deformation of wet olivine gouge reveals a slip-rate and state-dependent response at 200 ^°^C and a rate-dependent response at 300 ^°^C (82). (*B*) Dry olivine gouge shows a similar shift in deformation mechanism between 600 ^°^C and 1,000 ^°^C (78). (*C*) Basalt gouge exhibits a similar shift in behavior between 150 ^°^C and 500 ^°^C (89). The experimental data are shown in black, and the predictions using Eqs. **13** and **15** with the parameters in *SI Appendix*, Tables S4–S6 are shown in red (74, 82).

Consideration of the available mechanical data strongly suggests the involvement of multiple mechanisms of deformation within gouge layers, each characterized by different effective power-law exponents and thermodynamic parameters (47, 84). Typical mechanisms involved in cataclasis encompass granular flow, fracturing, and plastic flow in various proportions acting on different rock-forming minerals (80, 85, 86). The rise of the direct effect parameter is most dramatic across the transition from brittle to semi-brittle deformation, which is facilitated by temperature, but also by confining pressure and the presence of pressurized fluids. The transition from brittle to semi-brittle deformation is recognized in velocity-step experiments by a shift from a rate- and state-dependent response to an only rate-dependent response (Fig. 4). The mechanical response to velocity steps of dry olivine at 600 ^°^C and 1,000 ^°^C can be explained using two mechanisms of deformation with $n_{1}=80$ and $n_{2}=31$. Another transition from semi-brittle to ductile deformation at higher temperatures is apparent in the wet olivine experiments shown in Fig. 2*H*, which can be explained using three mechanisms of deformation with $n_{1}=110$, $n_{2}=19$, and $n_{3}=5$. Attaining the ductile conditions where pressure-solution, dislocation creep, dislocation-accommodated grain-boundary sliding, and diffusion creep dominate with power-law exponents n<10 is otherwise rare in the experimental conditions typically explored for frictional studies. Even halite, which can deform in the brittle, semi-brittle, and ductile regimes at room temperature, requires large power-law exponents for the shear stress dependence (87, 88).

The combined role of confining pressure and temperature on inducing the brittle-to-flow transition is perhaps most apparent in the velocity-step experiments conducted on phyllosilicates (Fig. 2*I*–*K*). At effective normal stress of 100 MPa, the direct effect parameter varies virtually independently of temperature, corresponding to the predominance of a single deformation mechanism. At 200 MPa and 300 MPa, a shift of mechanism occurs around 350 ^°^C that drastically increases the direct effect parameter, with relatively uniform values outside of the transition. A similar activation of semi-brittle deformation under high normal stress, wet conditions is seen for Westerly granite at high temperature, with a quasi-uniform direct effect parameter for dry granite at 5 MPa normal stress (Fig. 1*C*) and a transition to semi-brittle deformation above 350 ^°^C for wet granite under 400 MPa effective normal stress (Fig. 2*F*). These observations clearly demonstrate the progressive influence of semi-brittle deformation at high temperature as confining pressure rises. While the reference model seems inadequate to explain these observations, considering multiple mechanisms of deformation provides at least a qualitative interpretation.

## Conclusions

The specific physical assumptions that underlie a classic model of the slip-rate and state dependency of rock friction are undermined by laboratory observations. This realization forces a reassessment of the friction laws commonly employed in stress transfer analysis and seismic cycle simulations. A more realistic model should include the contributions of multiple mechanisms of deformation at frictional interfaces, encompassing the brittle to semi-brittle and the semi-brittle to ductile transitions. With the exception of granular flow, all deformation mechanisms associated with cataclasis are thermally activated, although not in the manner implied by (Eq. **5**). While there is an evolving understanding of the impacts of temperature, slip-rate, and effective normal stress on rock friction, further research is necessary to elucidate the additional controls of pore-fluid pressure and lithology. This is crucial to construct a comprehensive constitutive framework for fault mechanics.

## Methods

## Materials and Methods

The numerical procedure to simulate velocity and temperature steps experiments is described in *SI Appendix*, sections 1 and 2, respectively. Two separate derivations of the reference friction law are described in *SI Appendix*, section 3. The emergence of (Eq. **1**) and other empirical friction laws as a truncated Taylor series expansion of the alternative model is described in *SI Appendix*, section 4.

## Supplementary Material

Appendix 01 (PDF)

## Data Availability

Data (tables and Matlab scripts) have been deposited in Zenodo (90).

## References

[1] J. Byerlee, Friction of rock. Pure Appl. Geophys. **116**, 615–626 (1978).

[2] M. Blanpied, C. Marone, D. Lockner, J. Byerlee, D. King, Quantitative measure of the variation in fault rheology due to fluid-rock interactions. J. Geophys. Res. **103**, 9691–9712 (1998).

[3] J. Leeman, D. Saffer, M. Scuderi, C. Marone, Laboratory observations of slow earthquakes and the spectrum of tectonic fault slip modes. Nat. Commun. **7**, 11104 (2016). 27029996 10.1038/ncomms11104 PMC4821871

[4] D. M. Veedu , Bifurcations at the stability transition of earthquake faulting. Geophys. Res. Lett. **47**, e2020GL087985 (2020).

[5] E. Rabinowicz, The nature of the static and kinetic coefficients of friction. J. Appl. Phys. **22**, 1373–1379 (1951).

[6] J. H. Dieterich, Time-dependent friction in rocks. J. Geophys. Res. **77**, 3690–3697 (1972).

[7] J. H. Dieterich, Modeling of rock friction 1. Experimental results and constitutive equations. J. Geophys. Res. **84**, 2161–2168 (1979).

[8] A. Ruina, Slip instability and state variable friction laws. J. Geophys. Res. **88**, 10359–10370 (1983).

[9] S. T. Tse, J. R. Rice, Crustal earthquake instability in relation to the depth variation of frictional slip properties. J. Geophys. Res. **91**, 9452–9472 (1986).

[10] Y. Ben-Zion, J. R. Rice, Dynamic simulations of slip on a smooth fault in an elastic solid. J. Geophys. Res. **102**, 17771–17784 (1997).

[11] N. Lapusta, J. R. Rice, Y. BenZion, G. Zheng, Elastodynamics analysis for slow tectonic loading with spontaneous rupture episodes on faults with rate- and state-dependent friction. J. Geophys. Res. **105**, 23765–23789 (2000).

[12] N. Lapusta, Y. Liu, Three-dimensional boundary integral modeling of spontaneous earthquake sequences and aseismic slip. J. Geophys. Res. **114**, 25 (2009).

[13] S. Barbot, N. Lapusta, J. P. Avouac, Under the hood of the earthquake machine: Towards predictive modeling of the seismic cycle. Science **336**, 707–710 (2012). 22582259 10.1126/science.1218796

[14] Q. Qiu , The mechanism of partial rupture of a locked megathrust: The role of fault morphology. Geology **44**, 875–878 (2016).

[15] Y. Liu, J. R. Rice, Aseismic slip transients emerge spontaneously in three-dimensional rate and state modeling of subduction earthquake sequences. J. Geophys. Res. **110**, B08307 (2005).

[16] M. Wei, Y. Kaneko, Y. Liu, J. J. McGuire, Episodic fault creep events in California controlled by shallow frictional heterogeneity. Nat. Geosci. **6**, 566 (2013).

[17] B. Shibazaki, S. Bu, T. Matsuzawa, H. Hirose, Modeling the activity of short-term slow slip events along deep subduction interfaces beneath Shikoku, southwest Japan. J. Geophys. Res. **115**, B00A19 (2010).

[18] Q. Shi , Structural control and system-level behavior of the seismic cycle at the Nankai trough. Earth Planets Space **72**, 1–31 (2020).

[19] S. Barbot, Frictional and structural controls of seismic super-cycles at the Japan trench. Earth Planets Space **72**, 63 (2020).

[20] D. Veedu, S. Barbot, The Parkfield tremors reveal slow and fast ruptures on the same asperity. Nature **532**, 361–365 (2016). 27042936 10.1038/nature17190

[21] B. Wang, S. Barbot, Pulse-like ruptures, seismic swarms, and tremorgenic slow-slip events with thermally activated friction. Earth Planet. Sci. Lett. **603**, 117983 (2023).

[22] S. Barbot, Slow-slip, slow earthquakes, period-two cycles, full and partial ruptures, and deterministic chaos in a single asperity fault. Tectonophysics **768**, 228171 (2019).

[23] S. Nie, S. Barbot, Rupture styles linked to recurrence patterns in seismic cycles with a compliant fault zone. Earth Planet. Sci. Lett. **591**, 117593 (2022).

[24] C. Liang, J. P. Ampuero, D. Pino Muñoz, The paucity of supershear earthquakes on large faults governed by rate and state friction. Geophys. Res. Lett. **49**, e2022GL099749 (2022).

[25] H. Eyring, The activated complex in chemical reactions. J. Chem. Phys. **3**, 107–115 (1935).

[26] H. Eyring, Viscosity, plasticity, and diffusion as examples of absolute reaction rates. J. Chem. Phys. **4**, 283–291 (1936).

[27] J. Amuzu, B. Briscoe, D. Tabor, The shear properties of poly (n-alkyl methacrylates) in concentrated contacts. ASLE Trans. **20**, 152–160 (1977).

[28] R. Stesky, Mechanisms of high temperature frictional sliding in Westerly granite. Can. J. Earth Sci. **15**, 361–375 (1978).

[29] B. Briscoe, D. Evans, The shear properties of Langmuir-Blodgett layers. Proc. R. Soc. Lond. A Math. Phys. Sci. **380**, 389–407 (1982).

[30] F. Heslot, T. Baumberger, B. Perrin, B. Caroli, C. Caroli, Creep, stick-slip, and dry friction dynamics: Experiments and a heuristic model. Phys. Rev. E **49**, 4973–4988 (1994). 10.1103/physreve.49.4973 9961818

[31] Y. Brechet, Y. Estrin, The effect of strain rate sensitivity on dynamic friction of metals. Scr. Metall. Mater. **30**, 1449–1454 (1994).

[32] N. H. Sleep, Application of a unified rate and state dependent theory to the mechanics of fault zones with strain localization. J. Geophys. Res. **102**, 2875–2895 (1997).

[33] N. H. Sleep, Real contacts and evolution laws for rate and state friction. Geochem. Geophys. Geosyst. **7**, Q08012 (2006).

[34] T. Baumberger, P. Berthoud, C. Caroli, Physical analysis of the state- and rate-dependent friction law. II. Dynamic friction. Phys. Rev. B **60**, 3928–3939 (1999).

[35] M. Nakatani, Conceptual and physical clarification of rate and state friction: Frictional sliding as a thermally activated rheology. J. Geophys. Res. **106**, 13347–13380 (2001).

[36] T. Baumberger, C. Caroli, Solid friction from stick-slip down to pinning and aging. Adv. Phys. **55**, 279–348 (2006).

[37] N. Beeler, T. Tullis, A. Kronenberg, L. Reinen, The instantaneous rate dependence in low temperature laboratory rock friction and rock deformation experiments. J. Geophys. Res. **112**, B07310 (2007).

[38] T. Putelat, J. H. Dawes, J. R. Willis, On the microphysical foundations of rate-and-state friction. J. Mech. Phys. Solid **59**, 1062–1075 (2011).

[39] M. J. Ikari, B. M. Carpenter, C. Marone, A microphysical interpretation of rate-and state-dependent friction for fault gouge. Geochem. Geophys. Geosyst. **17**, 1660–1677 (2016).

[40] M. H. Linker, J. H. Dieterich, Effects of variable normal stress on rock friction: Observations and constitutive relations. J. Geophys. Res. **97**, 4923–4940 (1992).

[41] F. M. Chester, Effects of temperature on friction: Constitutive equations and experiments with fault gouge. J. Geophys. Res. **99**, 7247–7261 (1994).

[42] H. Noda, Frictional constitutive law at intermediate slip rates accounting for flash heating and thermally activated slip process. J. Geophys. Res. **113**, 12 (2008).

[43] Y. Bar-Sinai, R. Spatschek, E. A. Brener, E. Bouchbinder, On the velocity-strengthening behavior of dry friction. J. Geophys. Res. **119**, 1738–1748 (2014).

[44] J. Chen, C. J. Spiers, Rate and state frictional and healing behavior of carbonate fault gouge explained using microphysical model. J. Geophys. Res. **121**, 8642–8665 (2016).

[45] E. Aharonov, C. H. Scholz, A physics-based rock friction constitutive law: Steady state friction. J. Geophys. Res. **123**, 1591–1614 (2018).

[46] E. Aharonov, C. H. Scholz, The brittle-ductile transition predicted by a physics-based friction law. J. Geophys. Res. **124**, 2721–2737 (2019).

[47] J. Chen, A. Niemeijer, C. J. Spiers, Microphysical modeling of carbonate fault friction at slip rates spanning the full seismic cycle. J. Geophys. Res. **126**, e2020JB021024 (2021). 10.1029/2020JB021024 PMC8047899 33868888

[48] C. A. Thom, L. N. Hansen, D. L. Goldsby, E. E. Brodsky, A microphysical model of rock friction and the brittle-ductile transition controlled by dislocation glide and backstress evolution. J. Geophys. Res. **128**, e2022JB024150 (2023).

[49] C. Mei, J. W. Rudnicki, Microphysical modeling of fault slip and stability transition in hydrothermal conditions. Geophys. Res. Lett. **50**, e2023GL103730 (2023).

[50] J. R. Rice, N. Lapusta, K. Ranjith, Rate and state dependent friction and the stability of sliding between elastically deformable solids. J. Mech. Phys. Solid **49**, 1865–1898 (2001).

[51] J. H. Dieterich, B. D. Kilgore, Direct observation of frictional contacts: New insights for sliding memory effects. Pure Appl. Geophys. **143**, 283–302 (1994).

[52] J. H. Dieterich, B. D. Kilgore, Imaging surface contacts: Power law contact distributions and contact stresses in quartz, calcite, glass and acrylic plastic. Tectonophysics **256**, 219–239 (1996).

[53] N. H. Sleep, Physical basis of evolution laws for rate and state friction. Geochem. Geophys. Geosyst. **6**, Q11008 (2005).

[54] S. Barbot, Modulation of fault strength during the seismic cycle by grain-size evolution around contact junctions. Tectonophysics **765**, 129–145 (2019).

[55] N. M. Beeler, T. E. Tullis, J. D. Weeks, The roles of time and displacement in the evolution effect in rock friction. Geophys. Res. Lett. **21**, 1987–1990 (1994).

[56] J. P. Ampuero, A. M. Rubin, Earthquake nucleation on rate and state faults - aging and slip laws. J. Geophys. Res. **113**, B01302 (2008).

[57] A. P. Rathbun, C. Marone, Symmetry and the critical slip distance in rate and state friction laws. J. Geophys. Res. **118**, 3728–3741 (2013).

[58] P. Bhattacharya, A. M. Rubin, E. Bayart, H. M. Savage, C. Marone, Critical evaluation of state evolution laws in rate and state friction: Fitting large velocity steps in simulated fault gouge with time-, slip-, and stress-dependent constitutive laws. J. Geophys. Res. **120**, 6365–6385 (2015).

[59] P. Bhattacharya, A. M. Rubin, N. M. Beeler, Does fault strengthening in laboratory rock friction experiments really depend primarily upon time and not slip? J. Geophys. Res. **122**, 6389–6430 (2017).

[60] J. R. Rice, Y. Ben-Zion, Slip complexity in earthquake fault models. Proc. Nat. Acad. Sci. U.S.A. **93**, 3811–3818 (1996). 10.1073/pnas.93.9.3811 PMC39441 11607669

[61] B. Erickson , The community code verification exercise for simulating Sequences of Earthquakes and Aseismic Slip (SEAS). Seismol. Res. Lett. **91**, 874–890 (2020).

[62] J. Jiang , Community-driven code comparisons for three-dimensional dynamic modeling of sequences of earthquakes and aseismic slip. J. Geophys. Res. **127**, e2021JB023519 (2022).

[63] L. Dal Zilio, N. Lapusta, J. P. Avouac, T. Gerya, Subduction earthquake sequences in a non-linear visco-elasto-plastic megathrust. Geophys. J. Int. **229**, 1098–1121 (2022).

[64] P. Tian, C. He, Velocity weakening of simulated augite gouge at hydrothermal conditions: Implications for frictional slip of pyroxene-bearing mafic lower crust. J. Geophys. Res. **124**, 6428–6451 (2019).

[65] Y. Liu, C. He, Friction properties of hornblende and implications for slow-slip events in subduction zones. Tectonophysics **796**, 228644 (2020).

[66] E. Mitchell, Y. Fialko, K. Brown, Velocity-weakening behavior of Westerly granite at temperature up to 600 ^°^C. J. Geophys. Res. **121**, 6932–6946 (2016).

[67] M. An , Friction of Longmaxi shale gouges and implications for seismicity during hydraulic fracturing. J. Geophys. Res. **125**, e2020JB019885 (2020).

[68] S. den Hartog, M. Y. Thomas, D. Faulkner, How do laboratory friction parameters compare with observed fault slip and geodetically derived friction parameters? Insights from the Longitudinal Valley Fault Taiwan. J. Geophys. Res. **126**, e2021JB022390 (2021).

[69] R. Valdez II, H. Kitajima, D. Saffer, Effects of temperature on the frictional behavior of material from the Alpine Fault Zone, New Zealand. Tectonophysics **762**, 17–27 (2019).

[70] C. Boulton , Frictional properties of exhumed fault gouges in DFDP-1 cores, Alpine Fault, New Zealand. Geophys. Res. Lett. **41**, 356–362 (2014).

[71] D. E. Moore, D. A. Lockner, S. Hickman, Hydrothermal frictional strengths of rock and mineral samples relevant to the creeping section of the San Andreas Fault. J. Struct. Geol. **89**, 153–167 (2016).

[72] S. den Hartog, C. Marone, D. Saffer, Frictional behavior downdip along the subduction megathrust: Insights from laboratory experiments on exhumed samples at in situ conditions. J. Geophys. Res. **128**, e2022JB024435 (2023).

[73] S. Barbot, A rate-, state-, and temperature-dependent friction law with competing healing mechanisms. J. Geophys. Res. **127**, e2022JB025106 (2022).

[74] S. Barbot, Constitutive behavior of rocks during the seismic cycle. AGU Adv. **4**, e2023AV000972 (2023).

[75] S. Barbot, Transient and steady-state friction in non-isobaric conditions. Geochem. Geophys. Geosyst. **25**, e2023GC011279 (2024).

[76] S. Nie, S. Barbot, Velocity and temperature dependence of steady-state friction of natural gouge controlled by competing healing mechanisms. Geophys. Res. Lett. **51**, e2023GL106485 (2024).

[77] C. He, L. Luo, Q. M. Hao, Y. Zhou, Velocity-weakening behavior of plagioclase and pyroxene gouges and stabilizing effect of small amounts of quartz under hydrothermal conditions. J. Geophys. Res. **118**, 3408–3430 (2013).

[78] D. King, C. Marone, Frictional properties of olivine at high temperature with applications to the strength and dynamics of the oceanic lithosphere. J. Geophys. Res. **117**, B12203 (2012).

[79] S. den Hartog, A. Niemeijer, C. J. Spiers, New constraints on megathrust slip stability under subduction zone P-T conditions. Earth Planet. Sci. Lett. **353**, 240–252 (2012).

[80] M. L. Blanpied, D. A. Lockner, J. D. Byerlee, Frictional slip of granite at hydrothermal conditions. J. Geophys. Res. **100**, 13045–13064 (1995).

[81] L. Zhang, C. He, Y. Liu, J. Lin, Frictional properties of the South China Sea oceanic basalt and implications for strength of the Manila subduction seismogenic zone. Mar. Geol. **394**, 16–29 (2017).

[82] S. Barbot, L. Zhang, Constitutive behavior of olivine gouge across the brittle-ductile transition. Geophys. Res. Lett. **50**, e2023GL105916 (2023).

[83] L. Zhang, C. He, Frictional properties of phyllosilicate-rich mylonite and conditions for the brittle-ductile transition. J. Geophys. Res. **121**, 3017–3047 (2016).

[84] K. Chen , Cascading and pulse-like ruptures during the 2019 Ridgecrest earthquakes in the Eastern California Shear Zone. Nat. Commun. **11**, 1–8 (2020). 31911581 10.1038/s41467-019-13750-w PMC6946662

[85] C. He, Z. Wang, W. Yao, Frictional sliding of gabbro gouge under hydrothermal conditions. Tectonophysics **445**, 353–362 (2007).

[86] C. He, W. Tan, L. Zhang, Comparing dry and wet friction of plagioclase: Implication to the mechanism of frictional evolution effect at hydrothermal conditions. J. Geophys. Res. **121**, 6365–6383 (2016).

[87] T. Shimamoto, Transition between frictional slip and ductile flow for halite shear zones at room temperature. Science **231**, 711–714 (1986). 17800795 10.1126/science.231.4739.711

[88] H. Noda, T. Shimamoto, A rate- and state-dependent ductile flow law of polycrystalline halite under large shear strain and implications for transition to brittle deformation. Geophys. Res. Lett. **37**, L09310 (2010).

[89] H. Okuda, A. R. Niemeijer, M. Takahashi, A. Yamaguchi, C. J. Spiers, Hydrothermal friction experiments on simulated basaltic fault gouge and implications for megathrust earthquakes. J. Geophys. Res. **128**, e2022JB025072 (2023).

[90] S. Barbot, Data from “Does the direct effect of friction increase continuously with absolute temperature?”. Zenodo. 10.5281/zenodo.11215520. Deposited 19 May 2024. PMC11494312 39388270

