# Supplementary material for: Does the direct effect of friction increase continuously with absolute temperature?
Source: Proc Natl Acad Sci U S A. 2024 Oct 10;121(42):e2405111121. doi: 10.1073/pnas.2405111121 (PMC11494312; doi:10.1073/pnas.2405111121)
Supplement: Supplementary file 1 — Appendix 01 (PDF) [file pnas.2405111121.sapp.pdf]

Supporting Information for

Does the direct effect of friction increase continuously with absolute temperature?

Sylvain Barbot

E-mail: sbarbot@usc.edu

This PDF file includes:

Fig. S1
Tables S1 to S6
SI References

1. Numerical estimate of the direct effect parameter

To estimate the direct effect parameter, we conduct velocity-step experiments numerically assuming a slider assembly with an infinite stiffness. Starting with the steady-state strength at sliding velocity V_1, we impose an instantaneous jump to a sliding velocity V_2 at constant micro-asperity size. Based on the solutions at V_1 and V_2, we estimate the direct effect parameter

$$a = \frac{\partial \mu}{\partial \ln V} \qquad \text{[S1]}$$

by finite difference. We use the velocities V_1 and V_2 used in the respective laboratory experiments.

To calculate the steady-state size of micro-asperities at any temperature and velocity V_1, we solve for the steady-state micro-asperity size that satisfies Equation (14) or Equation (15) for $\dot{d} = 0$, providing

$$V_1 = \frac{2h}{\lambda} \sum_{k=1}^{N} \frac{G_k}{p_k d_{\text{ss}}^{p_k}} \left(\frac{\sigma}{\sigma_0} \right)^{q_k} \exp \left[-\frac{H_k}{R} \left(\frac{1}{T} - \frac{1}{T_k} \right) \right] . \qquad \text{[S2]}$$

As there is no closed-form solution, we find the root numerically. Equipped with the steady-state micro-asperity size d_{ss}, we solve numerically for the shear stress that satisfies

$$\begin{aligned} V_1 = & \sum_{k=1}^{M} V_0 \left(\frac{\tau_1}{\mu_0 \bar{\sigma}} \right)^{n_k} \left(\frac{d_{\text{ss}}}{d_0} \right)^{-\alpha n_k} \left(\frac{\sigma}{\sigma_0} \right)^{\beta n} \exp \left[-\frac{Q_k}{R} \left(\frac{1}{T} - \frac{1}{\bar{T}_k} \right) \right] \\ & + \sum_{k=M+1}^{M+P} V_0 \left(\frac{\tau_1}{\tau_0} \right)^{n_k} \exp \left[-\frac{Q_k}{R} \left(\frac{1}{T} - \frac{1}{\bar{T}_k} \right) \right] , \end{aligned} \qquad \text{[S3]}$$

which is obtained by combining Equations (10), (11), and (13) in the main text, and where $\mu_0 = \chi / \chi_n$ is the reference friction coefficient. We then calculate the new stress τ_2 caused by the new velocity V_2, which satisfies

$$\begin{aligned} V_2 = & \sum_{k=1}^{M} V_0 \left(\frac{\tau_2}{\mu_0 \bar{\sigma}} \right)^{n_k} \left(\frac{d_{\text{ss}}}{d_0} \right)^{-\alpha n_k} \left(\frac{\sigma}{\sigma_0} \right)^{\beta n} \exp \left[-\frac{Q_k}{R} \left(\frac{1}{T} - \frac{1}{\bar{T}_k} \right) \right] \\ & + \sum_{k=M+1}^{M+P} V_0 \left(\frac{\tau_2}{\tau_0} \right)^{n_k} \exp \left[-\frac{Q_k}{R} \left(\frac{1}{T} - \frac{1}{\bar{T}_k} \right) \right] , \end{aligned} \qquad \text{[S4]}$$

with the same micro-asperity size d_{ss}. The estimate of the direct effect parameter is

$$\tilde{a} = \frac{1}{\sigma} \frac{\tau_2 - \tau_1}{\ln V_2 / V_1} . \qquad \text{[S5]}$$

We use these estimates in Figures 1 and 2.

2. Temperature step experiments

To model temperature step experiments using the alternative model, we assume a spring-slider assembly with a finite stiffness k. Starting from steady-state at velocity V, we impose a step in temperature from θ_1 to θ_2, which is associated with a direct effect. We then solve for the evolution of frictional strength at the temperature θ_2 using a Runge-Kutta method. We assume that the change of temperature is instantaneous. In practice, it occurs gradually, but over an exceedingly small displacement, as the concurrent loading rate is 40 nm/s.

To calculate the steady-state size of micro-asperities at temperature θ_1 and velocity V, we solve numerically for the steady-state micro-asperity size d_{ss} that satisfies Equation (14) or Equation (15) for $\dot{d} = 0$, providing

$$V = \frac{2h}{\lambda} \sum_{k=1}^{N} \frac{G_k}{p_k d_{\text{ss}}^{p_k}} \left(\frac{\sigma}{\sigma_0} \right)^{q_k} \exp \left[-\frac{H_k}{R} \left(\frac{1}{\theta_1} - \frac{1}{T_k} \right) \right] . \qquad \text{[S6]}$$

We then solve for the steady-state strength τ_1 that satisfies

$$\begin{aligned} V = & \sum_{k=1}^{M} V_0 \left(\frac{\tau_1}{\mu_0 \bar{\sigma}} \right)^{n_k} \left(\frac{d_{\text{ss}}}{d_0} \right)^{-\alpha n_k} \left(\frac{\sigma}{\sigma_0} \right)^{\beta n} \exp \left[-\frac{Q_k}{R} \left(\frac{1}{\theta_1} - \frac{1}{\bar{T}_k} \right) \right] \\ & + \sum_{k=M+1}^{M+P} V_0 \left(\frac{\tau_1}{\tau_0} \right)^{n_k} \exp \left[-\frac{Q_k}{R} \left(\frac{1}{\theta_1} - \frac{1}{\bar{T}_k} \right) \right] . \end{aligned} \qquad \text{[S7]}$$

To evaluate the direct effect of the temperature jump, which is assumed instantaneous, we solve numerically for the stress τ_2 that satisfies

$$\begin{aligned} V = & \sum_{k=1}^{M} V_0 \left(\frac{\tau_1}{\mu_0 \bar{\sigma}}\right)^{n_k} \left(\frac{d_{ss}}{d_0}\right)^{-\alpha n_k} \left(\frac{\sigma}{\sigma_0}\right)^{\beta n} \exp\left[-\frac{Q_k}{R}\left(\frac{1}{\theta_2} - \frac{1}{\bar{T}_k}\right)\right] \\ & + \sum_{k=M+1}^{M+P} V_0 \left(\frac{\tau_1}{\tau_0}\right)^{n_k} \exp\left[-\frac{Q_k}{R}\left(\frac{1}{\theta_2} - \frac{1}{\bar{T}_k}\right)\right] \end{aligned} \quad \text{[S8]}$$

using the same size of micro-asperities, as it only changes gradually. When only one deformation mechanism and one healing mechanism operates, closed-form solutions can be used instead of numerical solutions. To model the evolutionary phase, we use a Runge-Kutta method to solve Equations (13) and (15) simultaneously. The constitutive parameters that reproduce the velocity and temperature step experiments on dry quartz gouge shown in Figure 3 are listed in Table S3.

3. Alternative derivations of the reference model

The reference model finds alternative expressions based on different assumptions about the effect of the state variable. In both cases, the direct effect derives from the stress dependence of the Gibbs free energy of sliding from absolute rate theory, giving rise to the same temperature dependence of the direct effect parameter $a = RT/\Omega\chi_n$. The different treatments of the state variable give rise to similar mathematical expressions in linearized form, but to different expressions in regularized form. We explain these slightly different models below.

A. Derivation based on the real area of contact. The first model is based on the modulation of the real area of contact by the state variable. The derivation is described in the main text, but summarized below for convenience. The frictional resistance results from the strength of a micro-asperity and the contact density (1)

$$\tau = \chi(V)\mathcal{A}(\theta) \ , \quad \text{[S9]}$$

where the plowing hardness χ is a function of slip-rate and the real area of contact density $\mathcal{A}$ is a function of the age of contact. In this model, the real area of contact density depends on apparent contact age as follows (1)

$$\mathcal{A}(\theta) = \frac{\sigma}{\chi_n}\left(1 + m \ln \frac{\theta V_0}{L}\right) \ , \quad \text{[S10]}$$

where σ is the effective normal stress, χ_n is the indentation hardness, and $m \ll 1$ is a constitutive parameter. Micro-asperity creep is thermally activated (2, 3) with a potential energy barrier modified by the influence of shear stress and random thermal fluctuations, leading to (1, 4–6)

$$V = V_0 \exp\left(-\frac{E - \chi\Omega}{RT}\right) \ , \quad \text{[S11]}$$

where E and Ω are the energy and volume of activation for a mole of constituent. Connecting Eqs. (S9-S11), and using the definition $\mu = \tau/\sigma$, we get the slip-rate and state-dependent friction law

$$\mu = \frac{1}{\chi_n}\left(\frac{E}{\Omega} + \frac{RT}{\Omega} \ln \frac{V}{V_0}\right)\left(1 + m \ln \frac{\theta V_0}{L}\right) \ . \quad \text{[S12]}$$

Neglecting the quadratic terms, we recognize the friction law

$$\mu = \mu_0 + a \ln \frac{V}{V_0} + b \ln \frac{\theta V_0}{L} \ , \quad \text{[S13]}$$

i.e., Eq. (1) in the main text, with $\mu_0 = E/\Omega\chi_n$, $a = RT/\Omega\chi_n$, and $b = m\mu_0$.

The friction laws of Eq. (S12), Eq. (S13), and Eq. (1) are ill-posed for vanishing velocities or stationary contact. A regularized version can be found by considering the probability of retrograde jump at low shear stress, with (4, 6, 7)

$$V = 2V_0 \exp\left(-\frac{E}{RT}\right) \sinh \frac{\chi\Omega}{RT} \ . \quad \text{[S14]}$$

By inverting Eq. (S14), we obtain the regularized slip-rate dependence of the plowing hardness

$$\chi(V) = \frac{RT}{\Omega} \sinh^{-1}\left[\frac{V}{2V_0} \exp\left(\frac{E}{RT}\right)\right] \ . \quad \text{[S15]}$$

Connecting Eqs. (S9), (S10), and (S15), we obtain the regularized slip-rate- and state-dependent friction law

$$\mu = \frac{RT}{\Omega\chi_n} \sinh^{-1}\left(\frac{V}{2V_0} \exp\left[\frac{E}{RT}\right]\right)\left(1 + m \ln \frac{\theta V_0}{L}\right) \ . \quad \text{[S16]}$$

Finally, using $\mu_0 = E/\Omega\chi_n$, $a = RT/\Omega\chi_n$, and $b = m\mu_0$, we can write

$$\mu = a \sinh^{-1}\left(\frac{V}{2V_0}\exp\frac{\mu_0}{a}\right)\left(1+\frac{b}{\mu_0}\ln\frac{\theta V_0}{L}\right) . \qquad \text{[S17]}$$

As we argue in the main text, the friction laws of Eqs. (S12) and (S16) are incompatible with the temperature dependence of the direct effect parameter for a wide range of rocks. The formulation is also inadequate to explain the evolution of the frictional resistance during temperature steps (8). The regularized forms of Eqs. (S16) and (S17) are the natural implications of the above assumptions. However, another regularized friction law is typically used in the literature. We describe the slightly different formulation below.

B. Energy-based derivation. The model described in this section derives from adhesion theory, which defines the friction coefficient — the ratio between the macroscopic shear and normal stress — as the ratio of the shear and normal strength at contact junctions (9)

$$\mu = \frac{\tau}{\sigma} = \frac{\chi(V)}{\chi_n} , \qquad \text{[S18]}$$

where χ and χ_n are the plowing and indentation hardness at the inferface, respectively. The model considers the thermal activation of micro-asperity creep

$$V = V_0 \exp\left[-\frac{E-\chi\Omega}{RT}\right] , \qquad \text{[S19]}$$

where E and Ω are the energy and volume of activation for a mole of constituent. Inverting Eq. (S19) leads to the velocity dependence of the plowing hardness

$$\chi(V) = \frac{E}{\Omega} + \frac{RT}{\Omega}\ln\frac{V}{V_0} . \qquad \text{[S20]}$$

The model then assumes that the activation energy depends on the age of contact (6, 10), following

$$E = E_0 + \Delta E \ln\frac{\theta V_0}{L} , \qquad \text{[S21]}$$

where E_0 is a reference activation energy and $\Delta E = \partial E/\partial \ln\theta$ controls the aging of the activation energy. Connecting Eqs. (S18-S21), we get the slip-rate- and state-dependent friction law

$$\mu = \frac{E_0}{\Omega\chi_n} + \frac{RT}{\Omega\chi_n}\ln\frac{V}{V_0} + \frac{\Delta E}{\Omega\chi_n}\ln\frac{\theta V_0}{L} . \qquad \text{[S22]}$$

We immediately recognize the friction law

$$\mu = \mu_0 + a\ln\frac{V}{V_0} + b\ln\frac{\theta V_0}{L} , \qquad \text{[S23]}$$

i.e., Eqs. (1) in the main text, with $\mu_0 = E_0/\Omega\chi_n$, $a = RT/\Omega\chi_n$, and $b = \Delta E/\Omega\chi_n$. We can write $b = m\mu_0$ with $m = \Delta E/E_0$.

The friction laws of Eqs. (S22) and (S23) are ill-posed for vanishing velocities or stationary contact. A regularized version emerges by considering the probability of retrograde jump at low shear stress, with (4, 6, 7)

$$V = 2V_0 \exp\left[-\frac{E}{RT}\right]\sinh\frac{\chi\Omega}{RT} . \qquad \text{[S24]}$$

By inverting Eq. (S24), we obtain a regularized expression of the slip-rate dependence of the plowing hardness

$$\chi(V) = \frac{RT}{\Omega}\sinh^{-1}\left(\frac{V}{2V_0}\exp\left[\frac{E}{RT}\right]\right) \qquad \text{[S25]}$$

Connecting Eqs. (S18), (S21), and (S25), we obtain the regularized slip-rate-, state-, and temperature-dependent friction law

$$\mu = \frac{RT}{\Omega\chi_n}\sinh^{-1}\left[\frac{V}{2V_0}\exp\left(\frac{E_0+\Delta E\ln\frac{\theta V_0}{L}}{RT}\right)\right] . \qquad \text{[S26]}$$

Finally, using $\mu_0 = E_0/\Omega\chi_n$, $a = RT/\Omega\chi_n$, and $b = \Delta E/\Omega\chi_n$, we can write

$$\mu = a\sinh^{-1}\left[\frac{V}{2V_0}\exp\left(\frac{\mu_0+b\ln\frac{\theta V_0}{L}}{a}\right)\right] , \qquad \text{[S27]}$$

which is Eq. (9) in the main text. Eq. (S27) is widely used in the seismic cycle modeling community (10–13). However, as we argue in the main text, the physical assumptions behind Eqs. (S22) and (S26) are not supported by available mechanical data, particularly the temperature dependence of the direct effect parameter a for a wide range of rocks. The functional form of Eq. (S22) is also incompatible with the mechanical data from temperature steps (8). Finally, the physical assumptions provide no insights on the normal stress dependence of the friction coefficient that is widely documented (14–27). Hence, the reference model is inadequate to explain the origin of friction, the evolution of the friction coefficient with normal-stress and temperature, and the temperature dependence of the effective frictional parameters.

The physical assumptions in the models presented in Section A and Section B predict the same temperature dependence of the direct effect parameter, i.e., $a = RT/\Omega\chi_n$. Hence, the data presented in the main text invalidates both flavors of the reference model.

4. Alternative model and simplified cases

Here, we describe the alternative model derivation and inspect the implications of various simplifying assumptions involving isobaric and isothermal conditions. The frictional resistance of natural surfaces is controlled by the real area of contact, which can be written (28–31)

$$\mathcal{A} = \frac{\sigma}{\chi_n}\left(\frac{d}{d_0}\right)^{\alpha}\left(\frac{\sigma}{\sigma_0}\right)^{-\beta}\,, \tag{S28}$$

where the size of contact d is a state variable subject to an evolution law and $d_0 = 1\,\mu\text{m}$ is a fixed reference size, σ and $\sigma_0 = 10\,\text{MPa}$ are the effective normal stress and a fixed reference value, respectively, and $\alpha \ll 1$ and $\beta \ll 1$ are power exponents. The possible nonlinear dependence on normal stress arises from the properties of Hertzian contacts, which can be rough, of various shapes, and randomized along the interface (31–35). At the macroscopic level, the yield strength emerges from the strength of micro-asperities and the density of contact,

$$\tau_y = \mathcal{A}\chi\,, \tag{S29}$$

where χ is the plowing hardness. A slip-rate, state, normal-stress, and temperature-dependent friction law can be obtained by assuming a thermally activated constitutive law for frictional sliding,

$$\frac{V}{V_0} = \left(\frac{\tau}{\tau_y}\right)^{n_1}\exp\left[-\frac{Q_1}{R}\left(\frac{1}{T}-\frac{1}{\bar{T}_1}\right)\right]\,, \tag{S30}$$

where Q_1 and $\bar{T}_1$ are the energy and temperature of activation, respectively, and $n_1 \gg 1$ is a power exponent. Combining Eqs. (S28-S30), we get the expression

$$\frac{V}{V_0} = \left(\frac{\tau}{\mu_0\sigma}\right)^{n_1}\left(\frac{d}{d_0}\right)^{-\alpha n_1}\left(\frac{\sigma}{\sigma_0}\right)^{\beta n_1}\exp\left[-\frac{Q_1}{R}\left(\frac{1}{T}-\frac{1}{\bar{T}_1}\right)\right]\,, \tag{S31}$$

where we have defined the reference friction coefficient

$$\mu_0 = \frac{\chi}{\chi_n}\,, \tag{S32}$$

compatible with the assumptions of adhesion theory (9), as in Eq. (S18). Eq. (S31) can be inverted to express shear stress as a function of the remaining variables, to obtain a slip-rate-, state-, normal-stress-, and temperature-dependent friction law in multiplicative form

$$\mu = \mu_0\left(\frac{V}{V_0}\right)^{\frac{1}{n_1}}\left(\frac{d}{d_0}\right)^{\alpha}\left(\frac{\sigma}{\sigma_0}\right)^{-\beta}\exp\left[\frac{Q_1}{n_1 R}\left(\frac{1}{T}-\frac{1}{\bar{T}_1}\right)\right]\,. \tag{S33}$$

Eq. (S33) simplifies considerably in isothermal and isobaric conditions with $T = T_0$ and $\sigma = \sigma_0$, giving

$$\mu = \mu_0\left(\frac{V}{V_0}\right)^{\frac{1}{n_1}}\left(\frac{d}{d_0}\right)^{\alpha}\,. \tag{S34}$$

An additive formulation based on logarithms akin to Eq. (1) in the main text can be obtained by a Taylor series expansion truncated to the linear terms of $\exp x$ and x^y, using $\exp x = 1 + x + \mathcal{O}(x^2)$ or $x^y = 1 + y\ln x + \mathcal{O}(\ln^2 x)$, which is justified by $1/n_1 \ll 1$ and $\alpha \ll 1$. The truncated Taylor series expansion of Eq. (S34) reads

$$\mu \approx \mu_0\left(1 + \frac{1}{n_1}\ln\frac{V}{V_0} + \alpha\ln\frac{d}{d_0}\right)\,, \tag{S35}$$

where we recognize the direct effect parameter $a = \mu_0/n_1 + \mathcal{O}(\ln V)$. The exact value of the direct effect parameter emerges from the definition

$$a = \frac{\partial\mu}{\partial\ln V} = \frac{\mu}{n_1}\,, \tag{S36}$$

which follows Eq. (17) in the main text in the case of a single mechanism of deformation, and can be approximated with $\mu \approx \mu_0 + \mathcal{O}(\ln V)$. Hence, the small variations of the direct effect parameter with temperature and slip-rate are associated with the respective change of the effective friction coefficient, as n_1 is constant. The emergence of a friction law in the mathematical form of Eq. (1) from a truncated Taylor series expansion of a different, more general expression explains the applicability of Eq. (1) for a wide range of physical conditions and materials. However, the coefficients involved in Eq. (1) are not constant, which limits the accuracy and predictive power of the formulation to a narrow range of slip-rates, temperatures, and pore-fluid and confining pressures. A more general expression, such as Eq. (13) in the main text, is required to capture the friction phenomenon in a wider range of conditions with constant constitutive parameters.

Relaxing the simplifying assumptions explains previous milestone improvements of the slip-rate- and state-dependent friction formalism (8, 36). In isobaric and non-isothermal conditions with $\sigma = \sigma_0$, Eq. (S33) simplifies to

$$\frac{V}{V_0} = \left(\frac{\tau}{\mu_0\sigma}\right)^{n_1}\left(\frac{d}{d_0}\right)^{-\alpha n_1}\exp\left[-\frac{Q_1}{R}\left(\frac{1}{T}-\frac{1}{\bar{T}_1}\right)\right]\,. \tag{S37}$$

A slip-rate-, state-, and temperature-dependent friction law in multiplicative form is obtained by inverting the relationship, to get

$$\mu = \mu_0 \left(\frac{V}{V_0}\right)^{\frac{1}{n_1}} \left(\frac{d}{d_0}\right)^{\alpha} \exp\left[\frac{Q_1}{n_1 R}\left(\frac{1}{T} - \frac{1}{\bar{T}_1}\right)\right] . \qquad \text{[S38]}$$

An additive form involving logarithms can be obtained by a truncated Taylor series expansion, providing

$$\mu = \mu_0 \left(1 + \frac{1}{n_1} \ln \frac{V}{V_0} + \alpha \ln \frac{d}{d_0} + \frac{Q_1}{n_1 R}\left[\frac{1}{T} - \frac{1}{\bar{T}_1}\right]\right) . \qquad \text{[S39]}$$

Eq. (S39) matches a previous formulation calibrated to temperature-step experiments (8), where the effect of temperature on friction is decoupled from the effect of velocity, allowing the explanation of velocity-step and temperature-step experiments with a single set of constitutive parameters.

In isothermal and non-isobaric conditions with $T = \bar{T}_1$, Eq. (S33) simplifies to

$$\mu = \mu_0 \left(\frac{V}{V_0}\right)^{\frac{1}{n_1}} \left(\frac{d}{d_0}\right)^{\alpha} \left(\frac{\sigma}{\sigma_0}\right)^{-\beta} , \qquad \text{[S40]}$$

highlighting the nonlinear dependence of the effective friction coefficient on normal stress. Upon a truncated Taylor series expansion, the slip-rate-, normal-stress-, and state-dependent friction law in additive form becomes

$$\mu = \mu_0 \left(1 + \frac{1}{n_1} \ln \frac{V}{V_0} + \alpha \ln \frac{d}{d_0} - \beta \ln \frac{\sigma}{\sigma_0}\right) , \qquad \text{[S41]}$$

where the direct effect of changes of normal stress on the frictional strength is explicit. Such formulation is compatible with a previously proposed expressions (36, 37), explaining simultaneously the direct and transient effects associated with normal stress perturbations (36, 38) and the strong normal stress dependence of the friction coefficient observed for a wide range of rocks (14–27, 31).

Hence, the alternative model unifies and expands previous formulations, providing a consistent framework to explain many laboratory observations, encompassing slip-rate, temperature, and normal stress steps conducted at different background slip-rates and temperatures. In addition, considering multiple deformation mechanisms, as in Eq. (13), captures the brittle-to-semi-brittle and semi-brittle-to-ductile transitions (39), providing a single expression to explain rock behavior throughout the crust at various stages of the seismic cycle.

The constitutive framework may explain all the frictional properties typically measured in the laboratory, including the effective friction coefficient, the direct and steady-state effects of slip-rate, and the characteristic weakening distance, as illustrated in Figure S1. The model also reproduces the detailed mechanical response to velocity steps at different temperatures, as shown in Figure 4 in the main text for wet Damaping olivine (39), dry San Carlos olivine (40), and basalt (41), with the constitutive parameters listed in Tables S4, S5, and S6, respectively.

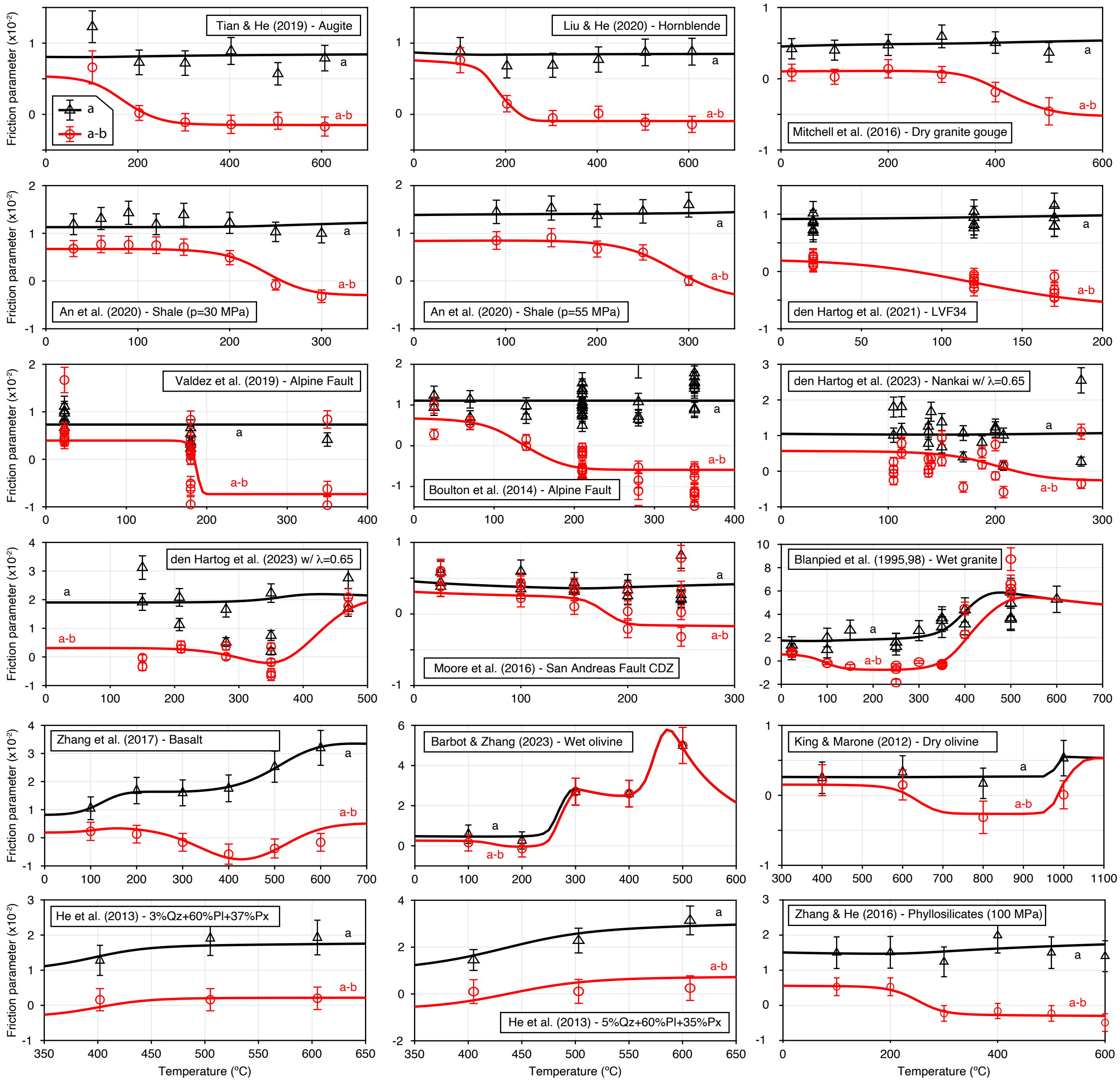

Fig. S1. Comparison between laboratory measurements and model predictions for the effective frictional parameters a and $a-b$ that describe the direct and steady-state effects of temperature, respectively. The alternative model explains the data for augite (42), hornblende (43), dry granite (44), wet granite (45, 46), shale (47), basalt (48), wet olivine (39), dry olivine (40), plagioclase and pyroxene (49), phyllosilicates (50), and natural samples from the Alpine Fault (51, 52), the Longitudinal Valley Fault (53), the Nankai subduction zone (54), and the Central Deforming Zone (CDZ) of the San Andreas Fault Observatory at Depth (SAFOD) (55).

Table S1. Thermodynamic parameters for a single deformation mechanism controlling the frictional response to velocity steps in isobaric condition for the rocks shown in Fig. 1. The scaling parameters are $V_0 = 1\,\mu$m/s, $d_0 = 1\,\mu$m, and $\sigma_0 = 10$ MPa.

Sample	n_1	Q_1 (kJ/mol)	$\bar{T}_1$ (°C)
Augite (42)	90	6	20
Hornblende (43)	85	6	20
Dry W. granite (44)	150	15	20
Shale (30 MPa) (47)	55	10	20
Shale (55 MPa) (47)	45	4	20
LVF34 (53)	60	15	20
Alpine Fault (52)	60	40	20
Alpine Fault (51)	55	55	20
Shimanto belt ($\lambda = 0.6$) (54)	70	25	20
Shimanto belt ($\lambda = 0.9$) (54)	40	25	20
SAFOD (55)	35	60	20

Table S2. Thermodynamic parameters of the deformation mechanisms controlling the direct effect parameters for various rocks shown in Fig. 2. The scaling parameters are $V_0 = 1$μm/s, $d_0 = 1$ μm, and $\sigma_0 = 10$ MPa. The dashes indicate only two deformation mechanisms.

Sample	n_1	n_2	n_3	$Q_1^\dagger$	$Q_2^\dagger$	$Q_3^\dagger$	$\bar{T}_1^\ddagger$	$\bar{T}_2^\ddagger$	$\bar{T}_3^\ddagger$
Dry albite (5)	75	23	-	50	65	-	20	290	-
	110	75	23	20	50	65	0	20	290
5%Qz mixture (49)	60	25	-	25	60	-	20	250	-
3%Qz mixture (49)	60	42	-	25	130	-	20	325	-
Dry olivine (40)	80	31	-	40	450	-	20	860	-
Metapelite (56)	60	7	-	20	105	-	20	250	-
Wet W. granite (46)	41	14	-	40	55	-	20	210	-
Basalt (48)	90	40	18	40	60	115	10	40	260
Wet olivine (39)	110	19	5	40	120	230	20	290	435

†Activation energy expressed in kJ/mol. ‡Activation temperature expressed in °C.

Table S3. Thermodynamic parameters for a single deformation mechanism controlling the frictional response of dry quartz gouge during velocity and temperature steps (Fig. 3). The constitutive behavior assumes $M = 1$ brittle mechanism and $P = 0$ semi-brittle mechanism and $N = 1$ healing mechanism. Considering the isobaric conditions, we take $\beta = 0$ and $q = 0$. †Scaling factor, not a proper constitutive parameter.

Parameter	Symbol	Value
Reference size of micro-asperities†	d_0	1 µm
Reference velocity†	V_0	1 µm/s
Reference growth rate for healing†	G_1	$(1\,\mu\text{m})^{p_1}$/s
Reference friction coefficient	μ_0	0.6827
Size dependency exponent	α	0.05
Stress power exponent	n_1	144
Reference temperature	$\bar{T}_1$	20°C
Size dependence exponent for healing	p_1	$2.1\alpha n_1$
Activation energy for healing	H_1	35 J/mol
Activation temperature for healing	T_1	20°C
Activation energy for direct effect	Q_1	55 kJ/mol
Reciprocal of characteristic strain	λ	1.75
effective normal stress	σ	20 MPa
Machine and sample stiffness	k	20 GPa/m
Shear zone thickness	h	0.5 mm

Table S4. Thermodynamic parameters explaining the mechanical response of wet Damaping olivine (39) upon velocity steps in isobaric conditions from 100°C to 500°C shown in Fig. 4a. The constitutive behavior requires $M = 1$ brittle mechanism and $P = 2$ semi-brittle mechanisms for a total of $M + P = 3$ deformation mechanisms and $N = 2$ healing mechanisms. Considering the isobaric conditions, we take $\beta = 0$ and $q = 0$. †Scaling factor, not a proper constitutive parameter. *The reference friction coefficient $\mu_0 = \chi/\chi_n$ is the ratio of the plowing to indentation hardness.

Parameter	Symbol	Value
Reference asperity size†	d_0	1 µm
Reference velocity†	V_0	1 µ/s
Reference healing rates†	G_1	$(1\,\mu\text{m})^{p_1}$
	G_2	$(1\,\mu\text{m})^{p_2}$
Reference friction coefficient*	μ_0	0.58
Micro-asperity size sensitivity	α	0.05
Stress power exponents	n_1	70
	n_2	17
	n_3	4
Activation energies for direct effect	Q_1	40 kJ/mol
	Q_2	240 kJ/mol
	Q_3	300 kJ/mol
Reference temperatures	$\bar{T}_1$	20°C
	$\bar{T}_2$	318°C
	$\bar{T}_3$	430°C
Healing power exponents	p_1	$2.2\,\alpha n_1$
	p_2	$0.9\,\alpha n_1$
Healing activation energies	H_1	10 kJ/mol
	H_2	70 kJ/mol
Healing activation temperatures	T_1	0°C
	T_2	180°C
Reciprocal of characteristic strain	λ	20
Effective normal stress	σ	90 MPa
Stiffness	k	50 GPa/m
Shear zone thickness	h	0.5 mm

Table S5. Thermodynamic parameters ([30]) explaining the mechanical response of dry San Carlos olivine ([40]) upon velocity steps in isobaric conditions from 400°C to 1,000°C shown in Fig. 4b. The constitutive behavior requires $M = 1$ brittle mechanism and $P = 2$ semi-brittle mechanisms for a total of $M + P = 3$ deformation mechanisms and $N = 2$ healing mechanisms. Considering the isobaric conditions, we take $\beta = 0$ and $q = 0$. †Scaling factor, not a proper constitutive parameter. *The reference friction coefficient $\mu_0 = \chi/\chi_n$ is the ratio of the plowing to indentation hardness.

Parameter	Symbol	Value
Reference asperity size†	d_0	1 µm
Reference velocity†	V_0	1 µ/s
Reference healing rates†	G_1	$(1\,\mu\text{m})^{p_1}$
	G_2	$(1\,\mu\text{m})^{p_2}$
Reference friction coefficient*	μ_0	0.5
Micro-asperity size sensitivity	α	0.05
Stress power exponents	n_1	80
	n_2	31
	n_3	4
Activation energies for direct effect	Q_1	40 kJ/mol
	Q_2	450 kJ/mol
	Q_3	500 kJ/mol
Reference temperatures	$\bar{T}_1$	20°C
	$\bar{T}_2$	860°C
	$\bar{T}_3$	1,450°C
Healing power exponents	p_1	$2.9\,\alpha n_1$
	p_2	$0.7\,\alpha n_1$
Healing activation energies	H_1	10 kJ/mol
	H_2	110 kJ/mol
Healing activation temperatures	T_1	0°C
	T_2	800°C
Reciprocal of characteristic strain	λ	20
Effective normal stress	σ	100 MPa
Stiffness	k	100 GPa/m
Shear zone thickness	h	1 mm

Table S6. Thermodynamic parameters (30) explaining the mechanical response of basalt (41) upon velocity steps in isobaric conditions from 100°C to 550°C shown in Fig. 4c. The constitutive behavior requires $M=1$ brittle mechanism and $P=1$ semi-brittle mechanisms for a total of $M+P=2$ deformation mechanisms and $N=2$ healing mechanisms. Considering the isobaric conditions, we take $\beta=0$ and $q=0$. †Scaling factor, not a proper constitutive parameter. *The reference friction coefficient $\mu_0=\chi/\chi_n$ is the ratio of the plowing to indentation hardness.

Parameter	Symbol	Value
Reference asperity size†	d_0	1 µm
Reference velocity†	V_0	1 µ/s
Reference healing rates†	G_1	$(1\,\mu\text{m})^{p_1}$
	G_2	$(1\,\mu\text{m})^{p_2}$
Reference friction coefficient*	μ_0	0.57
Micro-asperity size sensitivity	α	0.05
Stress power exponents	n_1	30
	n_2	30
Activation energies for direct effect	Q_1	40 kJ/mol
	Q_2	80 kJ/mol
Reference temperatures	$\bar{T}_1$	20°C
	$\bar{T}_2$	400°C
Healing power exponents	p_1	$2.1\,\alpha n_1$
	p_2	$0.8\,\alpha n_1$
Healing activation energies	H_1	35 kJ/mol
	H_2	40 kJ/mol
Healing activation temperatures	T_1	0°C
	T_2	75°C
Reciprocal of characteristic strain	λ	10
Effective normal stress	σ	100 MPa
Stiffness	k	300 GPa/m
Shear zone thickness	h	0.5 mm

References

1. T Baumberger, P Berthoud, C Caroli, Physical analysis of the state- and rate-dependent friction law. II. Dynamic friction. *Phys. Rev. B* **60**, 3928–3939 (1999).
2. H Eyring, The activated complex in chemical reactions. *The J. Chem. Phys.* **3**, 107–115 (1935).
3. H Eyring, Viscosity, plasticity, and diffusion as examples of absolute reaction rates. *The J. chemical physics* **4**, 283–291 (1936).
4. B Briscoe, D Evans, The shear properties of Langmuir—Blodgett layers. *Proc. royal society London. A. Math. physical sciences* **380**, 389–407 (1982).
5. M Nakatani, Conceptual and physical clarification of rate and state friction: Frictional sliding as a thermally activated rheology. *J. Geophys. Res.* **106**, 13347–13380 (2001).
6. JR Rice, N Lapusta, K Ranjith, Rate and state dependent friction and the stability of sliding between elastically deformable solids. *J. Mech. Phys. Solids* **49**, 1865–1898 (2001).
7. F Heslot, T Baumberger, B Perrin, B Caroli, C Caroli, Creep, stick-slip, and dry friction dynamics: experiments and a heuristic model. *Phys. Rev. E* **49**, 4973 – 4988 (1994).
8. FM Chester, Effects of temperature on friction: Constitutive equations and experiments with fault gouge. *J. Geophys. Res.* **99**, 7247–7261 (1994).
9. FP Bowden, D Tabor, *The friction and lubrication of Solids, Part I.* (Clarendon Press, Oxford), (1950).
10. P Segall, *Earthquake and volcano deformation.* (Princeton University Press, Princeton, NJ), (2010).
11. N Lapusta, Y Liu, Three-dimensional boundary integral modeling of spontaneous earthquake sequences and aseismic slip. *J. Geophys. Res.* **114**, 25 PP. (2009).
12. B Erickson, et al., The Community Code Verification Exercise for Simulating Sequences of Earthquakes and Aseismic Slip (SEAS). *Seism. Res. Lett.* (2020).
13. L Dal Zilio, N Lapusta, JP Avouac, T Gerya, Subduction earthquake sequences in a non-linear visco-elasto-plastic megathrust. *Geophys. J. Int.* **229**, 1098–1121 (2022).
14. J Handin, On the coulomb-mohr failure criterion. *J. Geophys. Res.* **74**, 5343–5348 (1969).
15. N Barton, Review of a new shear-strength criterion for rock joints. *Eng. geology* **7**, 287–332 (1973).
16. J Byerlee, Friction of rock. *Pure Appl. Geophys.* **116**, 615–626 (1978).
17. C Marone, JE Vidale, W Ellsworth, Fault healing inferred from the time dependent variations in source properties of repeating earthquakes. *Geophys. Res. Lett.* **22**, 3095–3098 (1995).
18. DE Moore, DA Lockner, H Tanaka, K Iwata, The coefficient of friction of chrysotile gouge at seismogenic depths. *Int. Geol. Rev.* **46**, 385–398 (2004).
19. DE Moore, DA Lockner, Friction of the smectite clay montmorillonite: A review and interpretation of data. *The seismogenic zone subduction thrust faults* pp. 317–345 (2007).
20. DE Moore, DA Lockner, Talc friction in the temperature range 25°-400°C: Relevance for fault-zone weakening. *Tectonophysics* **449**, 120–132 (2008).
21. SA den Hartog, CJ Peach, DM de Winter, CJ Spiers, T Shimamoto, Frictional properties of megathrust fault gouges at low sliding velocities: New data on effects of normal stress and temperature. *J. Struct. Geol.* **38**, 156–171 (2012).
22. S Maegawa, F Itoigawa, T Nakamura, Effect of normal load on friction coefficient for sliding contact between rough rubber surface and rigid smooth plane. *Tribol. international* **92**, 335–343 (2015).
23. S Yashima, et al., Normal contact and friction of rubber with model randomly rough surfaces. *Soft Matter* **11**, 871–881 (2015).
24. CA Morrow, DE Moore, DA Lockner, Frictional strength of wet and dry montmorillonite. *J. Geophys. Res. Solid Earth* **122**, 3392–3409 (2017).
25. MM Scuderi, BM Carpenter, Frictional stability and hydromechanical coupling of serpentinite-bearing fault gouge. *Geophys. J. Int.* **231**, 290–305 (2022).
26. Ki Hirauchi, R Hibi, R Shirahige, T Takemura, Effects of phyllosilicate content on the slip behavior of fault gouge: Insights from room-temperature friction experiments on quartz–talc mixtures. *Tectonophysics* **857**, 229845 (2023).
27. G Pozzi, et al., Fabric controls fault stability in serpentinite gouges. *Geophys. J. Int.* **235**, 1778–1797 (2023).
28. S Barbot, Modulation of fault strength during the seismic cycle by grain-size evolution around contact junctions. *Tectonophysics* **765**, 129–145 (2019).
29. S Barbot, A rate-, state-, and temperature-dependent friction law with competing healing mechanisms. *J. Geophys. Res.* **127**, e2022JB025106 (2022).
30. S Barbot, Constitutive behavior of rocks during the seismic cycle. *AGU Adv.* **4** (2023).
31. S Barbot, Transient and steady-state friction in non-isobaric conditions. *Geochem. Geophys. Geosystems* **25**, e2023GC011279 (2024).
32. H Hertz, Über die Berührung fester elastischer Körper. *J. für die reine und angewandte Math.* **92**, 156–171 (1881).
33. JF Archard, Elastic deformation and the laws of friction. *Proc. R. Soc. Lond. A* **243**, 190–205 (1957).
34. JA Greenwood, J Williamson, Contact of nominally flat surfaces. *Proc. R. Soc. London, Ser. A.,* **295**, 300–319 (1966).
35. NH Sleep, Real contacts and evolution laws for rate and state friction. *Geochem. Geophys. Geosystems* **7** (2006).
36. MH Linker, JH Dieterich, Effects of variable normal stress on rock friction: Observations and constitutive relations. *J. Geophys. Res.* **97**, 4923–4940 (1992).

37. NH Sleep, Physical basis of evolution laws for rate and state friction. *Geochem. Geophys. Geosystems* **6** (2005).
38. T Hong, C Marone, Effects of normal stress perturbations on the frictional properties of simulated faults. *Geochem. Geophys. Geosystems* **6** (2005).
39. S Barbot, L Zhang, Constitutive behavior of olivine gouge across the brittle-ductile transition. *Geophys. Res. Lett.* **50** (2023).
40. D King, C Marone, Frictional properties of olivine at high temperature with applications to the strength and dynamics of the oceanic lithosphere. *J. Geophys. Res.* **117** (2012).
41. H Okuda, AR Niemeijer, M Takahashi, A Yamaguchi, CJ Spiers, Hydrothermal friction experiments on simulated basaltic fault gouge and implications for megathrust earthquakes. *J. Geophys. Res.* **128**, e2022JB025072 (2023).
42. P Tian, C He, Velocity weakening of simulated augite gouge at hydrothermal conditions: Implications for frictional slip of pyroxene-bearing mafic lower crust. *J. Geophys. Res.* **124**, 6428–6451 (2019).
43. Y Liu, C He, Friction properties of hornblende and implications for slow-slip events in subduction zones. *Tectonophysics* **796**, 228644 (2020).
44. E Mitchell, Y Fialko, K Brown, Velocity-weakening behavior of Westerly granite at temperature up to 600°C. *J. Geophys. Res.* **121**, 6932–6946 (2016).
45. ML Blanpied, DA Lockner, JD Byerlee, Frictional slip of granite at hydrothermal conditions. *J. Geophys. Res.* **100**, 13045–13064 (1995).
46. M Blanpied, C Marone, D Lockner, J Byerlee, D King, Quantitative measure of the variation in fault rheology due to fluid-rock interactions. *J. Geophys. Res.* **103**, 9691–9712 (1998).
47. M An, et al., Friction of Longmaxi shale gouges and implications for seismicity during hydraulic fracturing. *J. Geophys. Res.* **125**, e2020JB019885 (2020).
48. L Zhang, C He, Y Liu, J Lin, Frictional properties of the South China Sea oceanic basalt and implications for strength of the Manila subduction seismogenic zone. *Mar. Geol.* **394**, 16–29 (2017).
49. C He, L Luo, QM Hao, Y Zhou, Velocity-weakening behavior of plagioclase and pyroxene gouges and stabilizing effect of small amounts of quartz under hydrothermal conditions. *J. Geophys. Res.* **118**, 3408–3430 (2013).
50. L Zhang, C He, Frictional properties of phyllosilicate-rich mylonite and conditions for the brittle-ductile transition. *J. Geophys. Res.* **121**, 3017–3047 (2016).
51. C Boulton, et al., Frictional properties of exhumed fault gouges in DFDP-1 cores, Alpine Fault, New Zealand. *Geophys. Res. Lett.* **41**, 356–362 (2014).
52. R Valdez II, H Kitajima, D Saffer, Effects of temperature on the frictional behavior of material from the Alpine Fault Zone, New Zealand. *Tectonophysics* **762**, 17–27 (2019).
53. S den Hartog, MY Thomas, D Faulkner, How do laboratory friction parameters compare with observed fault slip and geodetically derived friction parameters? Insights from the Longitudinal Valley Fault, Taiwan. *J. Geophys. Res.* **126**, e2021JB022390 (2021).
54. S den Hartog, C Marone, D Saffer, Frictional behavior downdip along the subduction megathrust: Insights from laboratory experiments on exhumed samples at in situ conditions. *J. Geophys. Res.* **128**, e2022JB024435 (2023).
55. DE Moore, DA Lockner, S Hickman, Hydrothermal frictional strengths of rock and mineral samples relevant to the creeping section of the San Andreas Fault. *J. Struct. Geol.* **89**, 153–167 (2016).
56. S den Hartog, A Niemeijer, CJ Spiers, New constraints on megathrust slip stability under subduction zone P–T conditions. *Earth Planet. Sci. Lett.* **353**, 240–252 (2012).
